# A cohort study of circulating progenitor cells after ST-segment elevation and non-ST segment elevation myocardial infarction in non-diabetic and diabetic patients

**DOI:** 10.3389/fcvm.2022.1011140

**Published:** 2022-11-17

**Authors:** Andreas Baumbach, Yu-Xin Cui, Rebecca N. Evans, Lucy Culliford, Tom Johnson, Chris A. Rogers, Barnaby C. Reeves, Chiara Bucciarelli-Ducci, Jessica Harris, Mark Hamilton, Paolo Madeddu

**Affiliations:** ^1^Bristol Heart Institute, University of Bristol, Bristol, United Kingdom; ^2^William Harvey Research Institute, Queen Mary University of London, London, United Kingdom; ^3^Bristol Trials Centre, Bristol Medical School, University of Bristol, Bristol, United Kingdom; ^4^Royal Brompton and Harefield Hospitals, Guys’ and St.Thomas NHS Hospitals and School of Biomedical Engineering & Imaging Sciences, King’s College London, London, United Kingdom

**Keywords:** progenitor cells, migration, myocardial infarction, cardiac MRI, diabetes mellitus

## Abstract

**Background:**

Myocardial infarction induces elevation of progenitor cells in the circulation, a reparative response inhibited by type-2 diabetes.

**Objectives:**

Determine if myocardial infarct severity and diabetes interactively influence the migratory activity of CD34+/CXCR4+ progenitor cells and if the migratory test predicts cardiac outcomes.

**Materials and methods:**

A longitudinal study was conducted on patients with or without diabetes with a STEMI or NSTEMI. CD34+/CXCR4+ cells were measured in the peripheral blood using flow cytometry, and migratory activity was tested *in vitro* on cells isolated from samples collected on days 0 and 4 post-infarct. Cardiac function was assessed at three months using cardiac MRI.

**Results:**

Of 1,149 patients screened, 71 (6.3%) were eligible and consented. Fifty had STEMI (16 with diabetes) and 21 NSTEMI (8 with diabetes). The proportion of CD34+/CXCR4+ cells within blood mononuclear cells was 1.96 times higher after STEMI compared with NSTEMI (GMR = 1.96, 95% CI 0.87, 4.37) and 1.55 times higher in patients with diabetes compared to patients without diabetes (GMR = 1.55, 95% CI 0.77, 3.13). In the latter, STEMI was associated with a 2.42-times higher proportion of migrated CD34 + /CXCR4 + cells compared with NSTEMI (GMR = 2.42, 95% CI 0.66, 8.81). In patients with diabetes, the association was the opposite, with a 55% reduction in the proportion of migrated CD34+/CXCR4+ cells. No statistically significant associations were observed between the frequency in peripheral blood or *in vitro* migration capacity of CD34+/CXCR4+ cells and MRI outcomes.

**Conclusion:**

We document the interaction between infarct and diabetes on the migratory activity of CD34+/CXCR4+ cells. The test did not predict functional outcomes in the studied cohort.

## Background

Tissue repair after myocardial infarction (MI) comprises a phase dominated by the recruitment of neutrophils and proinflammatory macrophages and a subsequent resolution stage orchestrated by reparative macrophages (1–3). Progenitor cells (PCs) are also released from the bone marrow (BM) into the circulation and recruited to the injured myocardium through chemokine-orchestrated homing. Flow cytometry and *in vitro* migratory assays make it possible to define specific sub-populations of circulating PCs (CPCs) in relation to their functional properties (4).

The proportion of CPCs increases during the first week after the onset of acute MI (5–7), with an intensity not necessarily proportional to the increase in leukocyte counts (5). This finding suggests that the liberation of CPCs is a critical, autonomous determinant of post-MI healing. We and others have shown that type-2 diabetes mellitus (DM) and cardiovascular risk factors can impair the migratory and functional capacities of CPCs by negatively influencing the BM microenvironment (8–15), and the molecular crosstalk between mobilized cells and peripheral vasculature (16–18). CPC deficits have been claimed to represent excellent long-term predictors of cardiovascular outcomes in patients with type 2 DM (19–21). Moreover, new anti-diabetic drugs, such as glucagon-like peptide-1 receptor agonists and DPP-4 inhibitors, which reduce the risk of cardiovascular death in patients with DM (22, 23), increase the number and function of CPCs in peripheral blood (24–27). Therefore, assessing the state of CPCs could have prognostic and therapeutic implications.

It remains, however, unknown whether the negative effect of DM on CPC mobilization is influenced by the amount of damage to the heart after an acute MI and whether this interaction affects the post-MI outcome. To explore these questions, we have conducted a longitudinal study on patients with or without DM who have had an ST-segment Elevation MI (STEMI) or Non-ST-segment Elevation MI (NSTEMI). The first objective was to determine the interactive influence between DM and type of MI on (i) the relative abundance of CD34+/CXCR4+CPCs in peripheral blood (PB) and (ii) the *in vitro* migratory response of CPCs. The second objective was to investigate whether CPC abundance and migratory activity predicted cardiac magnetic resonance imaging (MRI) outcomes three months post-MI.

## Materials and methods

### Study design

ProMIS was a single center longitudinal, observational study conducted in Bristol, comprising four cohorts: patients with and without DM who have had a STEMI or a NSTEMI, defined according to international guidelines (28, 29). The study schema is illustrated in Supplementary Figure 1 and the study protocol in Supplementary Appendix 1. The study protocol and subsequent amendments received a favorable opinion from the Wiltshire Research Ethics Committee (ref 09/H0104/58) and were performed according to the declaration of Helsinki. The University Hospitals Bristol NHS Foundation Trust (UHBristol) was the sponsor. This study was overseen by the Cardiovascular Research Board of UHBristol and the University of Bristol.

### Objectives

The specific study objectives were to determine whether: (1a) the number of CPCs differs after STEMI compared to NSTEMI; (1b) the migratory capacity of CPCs varies in patients with or without DM; (1c) the number and migratory ability of CPCs are associated with covariables characterizing the severity of the initial STEMI or NSTEMI (e.g., troponin I, high sensitivity C-reactive protein (hs-CRP) or the quality of glucose control (HbA1c); and (2) the number and migratory capacity of CPCs after a STEMI or NSTEMI influence the size of the myocardial scar and myocardial contractility three months after the initial cardiac event.

### Participants

Patients aged 40–75 at admission with STEMI or NSTEMI presenting to a Bristol Heart Institute cardiologist within 24 h after the onset of symptoms and willing to participate for the duration of the study were eligible for inclusion. Patients with anemia (i.e., hemoglobin < 10 mg/dl), cardiogenic shock on presentation, renal impairment (eGFR < 50 ml), haemodynamic instability, contraindications to having the MRI scan (e.g., metallic implant, pacemakers, screws, claustrophobia, etc.), a previous coronary event within the last 12 weeks, participation in another clinical study, unable or unwilling to return for follow-up by the study schedule after three months or heightened anxiety during recruitment were excluded.

### Study procedures and follow-up

Patients with STEMI underwent immediate emergency intervention in the catheterization laboratory. Patients with NSTEMI with no ongoing symptoms or signs of ischemia underwent diagnostic angiography the following working day.

Patients were approached for enrollment into the study on day 0, at the time of PCI or diagnostic angiography. Blood samples were obtained within 12 h of presentation. A research MRI scan was scheduled before discharge, usually between days 2 and 4. A second MRI scan was scheduled at three months. A summary of time-points of data collection is provided in Supplementary Table 1.

### Laboratory measurements

The standardized protocol for counting CPCs and assessing their migratory activity has been described by us earlier (4).

### Cardiac magnetic resonance imaging scans

All MRI scans were performed on a 1.5T scanner (Magnetom Avanto; Siemens; Elangen, Germany), with a surface phased array received coil and retrospective electrographic triggering. Myocardial perfusion and previous infarction were assessed using adenosine and gadolinium-DTPA first-pass data acquisition and late-enhancement imaging.

Regional LV thickening of the “affected” segments was determined from end-systolic and end-diastolic images. Secondary measures of wall thickening included standard wall score index (WMSI); wall motion for each segment was rated from cine MRI images on a 5-point scale (0, normal; 1, mild hypokinesia; 2, severe hypokinesia; 3, akinesia; 4, dyskinesia).

LV regional myocardial viability was measured using gadolinium contrast images. Infarct size was measured as a planimeter area (and volume derived by multiplying the area by slice thickness) and volume fraction of enhanced myocardium. For myocardial viability analysis, delayed-enhancement images for each segment were scored for function analysis, and quantified by computer-assisted planimetry on short-axis images. Segments were graded in transmural extent on a 5-point scale (0, no HE; 1, hyper-enhancement extending from 1 to 25%; 2, 26 to 50%; 3, 51 to 75%; 4, hyperenhancement >75% of LV wall thickness for that segment).

End systolic volume, stroke volume and ejection fraction were measured according to the validated MRI laboratory standards, using contiguous short axis slices obtained by cine MRI with correction for long axis motion (Argus 4D Software or Brompton). End-diastolic and end-systolic endocardial traces were used to determine end-diastolic and end-systolic LV volumes and total ejection fraction.

### Statistical analysis

Baseline characteristics are summarized by exposure (STEMI and NSTEMI, without DM/DM) for all participants in the analysis population. Continuous variables are summarized using the mean and standard deviation (SD) or median and inter-quartile range (IQR) if distributions are skewed, and categorical data are summarized as a number and percentage.

The effect of the STEMI/NSTEMI and DM on CPC migration was estimated using a continuous longitudinal mixed effects methodology. All models incorporated random intercept and slope components where appropriate and used restricted maximum likelihood (REML) estimation and Kenward-Roger estimators to adjust the bias of estimating standard errors in small samples. Model fit was assessed using standard methods, such as residual plots and normality tests. If the assumptions were not met, outcomes were analyzed on a logarithmic scale with results presented as geometric mean ratios.

Two exposures of interest were considered for assessing the predictive value of CPCs: (1) the proportion of CD34+/CXCR4+ measured in the PB (within-person mean of replicates on day 0 and day 4) and (2) the ratio of migrated CD34+/CXCR4+ measured after STEMI or NSTEMI. Continuous outcomes were compared using linear regression and binary outcomes using logistic regression, adjusted for age, sex, time to MRI and baseline value of outcomes being modeled. For hypothesis tests, a two-tailed significance level of 5% was used, except for interactions. A 10% significance level was used to test the interactions between STEMI and DM, STEMI and timepoint, DM and timepoint, STEMI and chemo-attractant and DM and chemo-attractant. All analyses were performed in Stata version 17⋅0 (StataCorp, LP, College Station, TX, USA).

## Results

### Recruitment

**FIGURE 1:**
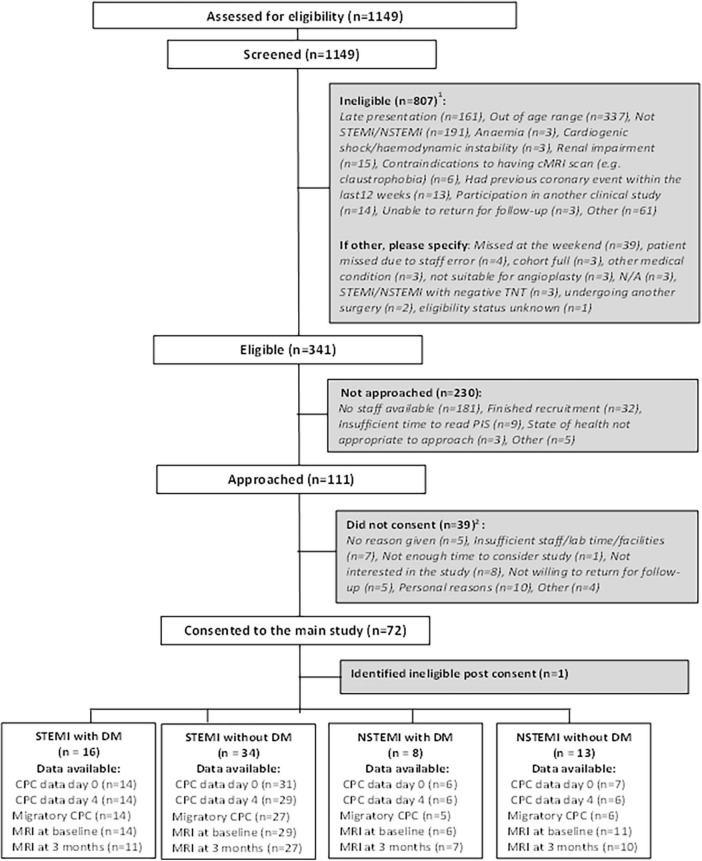
CONSORT diagram showing the flow of participants. Some patients may be ineligible for more than one reason. Some patients may have not consented for more than one reason.

Between February 2010 and September 2013, 1,149 patients were screened for inclusion. Of these, 807 (70.2%) were ineligible, 230 (20.0%) were eligible but not approached and 111 (9.7%) were eligible and approached for consent. Of the 111 patients approached for consent, 72 consented (6.3% of screened patients), of which one was identified as ineligible post-consent. Of the 71 consented participants, 50 were STEMI (16 with DM, 34 without DM) and 21 were NSTEMI (8 with DM, 13 without DM). The CONSORT diagram is presented in full in Figure 1.

### Patient withdrawals

Two participants withdrew from the study (1 STEMI without DM, 1 NSTEMI without DM). Details of the withdrawals are given in Supplementary Table 2.

### Protocol deviations

There were 32 protocol deviations (6 STEMI with DM, 9 STEMI without DM, 6 NSTEMI with DM, and 11 STEMI without DM). A summary of the protocol deviations is presented in Supplementary Table 3.

### Baseline characteristics

All 71 consented participants had baseline data available and had a preoperative angiographic assessment. Patient characteristics are presented in Table 1. The median age of participants was 61.0 years (IQR 52.7, 66.6). The median age was highest in the group of participants with NSTEMI and DM (68.0 years, IQR 57.9, 72.1) and similar across the remaining groups.

**TABLE 1:** Patient demography and past history.

Characteristic		STEMI with DM (*n* = 16)	STEMI without DM (*n* = 34)	NSTEMI with DM (*n* = 8)	NSTEMI without DM (*n* = 13)	Overall (*n* = 71)
**Patient demographics**						
Gender	Females	4/16(25.0%)	5/34(14.7%)	0/8(0.0%)	3/13(23.1%)	12/71(16.9%)
	Males	12/16(75.0%)	29/34(85.3%)	8/8(100.0%)	10/13(76.9%)	59/71(83.1%)
Age	Median (IQR) years	60.5(53.0,64.5)	59.5(52.0,66.0)	68.0(57.9,72.1)	58.0(52.0,62.0)	61.0(52.7,66.6)
Type of diabetes	Type I	4/11(36.4%)	0/34(0.0%)	5/7(71.4%)	0/13(0.0%)	12/18(66.7%)
	Type II	7/11(63.6%)	0/34(0.0%)	1/7(14.3%)	0/13(0.0%)	5/18(27.8%)
	Type III	0/11(0.0%)	0/34(0.0%)	1/7(14.3%)	0/13(0.0%)	1/18(5.6%)
BMI^a^	Median (IQR)	30.5(25.8,33.7)	26.3(22.3,29.4)	28.4(26.1,31.2)	27.7(24.8,29.7)	27.3(24.3,30.5)
Heartrate	Mean (SD) bpm	76.9 (16.2)	73.5 (17.7)	89 (21.9)	73.7 (16.0)	76 (17.8)
Systolic blood pressure	Mean (SD) mmHg	139.2 (22.2)	129.6 (26.7)	130 (24.5)	138.4 (17.5)	133 (24.0)
Diastolic blood pressure	Mean (SD) mmHg	81.9 (15.4)	82.4 (24.6)	76 (11.8)	86.5 (29.8)	82 (22.6)
Smoking history	Non-smoker	7/16(43.8%)	8/34(23.5%)	1/8(12.5%)	3/13(23.1%)	19/71(26.8%)
	Current smoker	2/16(12.5%)	19/34(55.9%)	2/8(25.0%)	8/13(61.5%)	31/71(43.7%)
	Ex-smoker	7/16(43.8%)	7/34(20.6%)	5/8(62.5%)	2/13(15.4%)	21/71(29.6%)
**Medical history**						
Family history of heart disease		10/16(62.5%)	15/33(45.5%)	5/8(62.5%)	5/11(45.5%)	35/68(51.5%)
Hypercholesterolaemia		13/16(81.3%)	11/34(32.4%)	3/8(37.5%)	5/13(38.5%)	32/71(45.1%)
Hypertension		11/16(68.8%)	9/34(26.5%)	5/8(62.5%)	8/13(61.5%)	33/71(46.5%)
Chronic pulmonary disease		0/16(0.0%)	3/34(8.8%)	0/8(0.0%)	0/13(0.0%)	3/71(4.2%)
Peripheral vascular disease		1/16(6.3%)	0/34(0.0%)	0/8(0.0%)	0/13(0.0%)	1/71(1.4%)
Hypothyroidism/hyperthyroidism		2/16(12.5%)	0/34(0.0%)	0/8(0.0%)	0/13(0.0%)	2/71(2.8%)
Cerebrovascular disease		0/16(0.0%)	0/34(0.0%)	1/8(12.5%)	0/13(0.0%)	1/71(1.4%)
Renal failure		0/16(0.0%)	1/34(2.9%)	0/8(0.0%)	0/13(0.0%)	1/71(1.4%)
Previous MI		3/16(18.8%)	4/34(11.8%)	0/8(0.0%)	1/13(7.7%)	8/71(11.3%)
Previous PCI		3/16(18.8%)	4/34(11.8%)	0/8(0.0%)	2/13(15.4%)	9/71(12.7%)
Previous CABG		0/16(0.0%)	0/34(0.0%)	0/8(0.0%)	1/13(7.7%)	1/71(1.4%)
**Pre-morbid symptom status**						
NYHA Classification	No symptoms/ limitations in PA	15/16(93.8%)	26/32(81.3%)	6/7(85.7%)	8/12(66.7%)	55/67(82.1%)
	Mild symptoms and slight limitations	1/16(6.3%)	5/32(15.6%)	1/7(14.3%)	4/12(33.3%)	11/67(16.4%)
	Marked limitation due to symptoms	0/16(0.0%)	1/32(3.1%)	0/7(0.0%)	0/12(0.0%)	1/67(1.5%)
CCS Classification	Asymptomatic	15/15(100.0%)	27/32(84.4%)	7/7(100.0%)	8/12(66.7%)	57/66(86.4%)
	Angina with prolonged exertion	0/15(0.0%)	2/32(6.3%)	0/8(0.0%)	2/12(16.7%)	4/66(6.1%)
	Slight limitation in activity	0/15(0.0%)	1/32(3.1%)	0/8(0.0%)	1/12(8.3%)	2/66(3.0%)
	Marked limitation of activity	0/15(0.0%)	2/32(6.3%)	0/8(0.0%)	1/12(8.3%)	3/66(4.5%)
**Medications on admission**						
Aspirin		8/16(50.0%)	10/34(29.4%)	3/8(37.5%)	2/13(15.4%)	23/71(32.4%)
Clopidogrel		2/16(12.5%)	5/34(14.7%)	1/8(12.5%)	1/13(7.7%)	9/71(12.7%)
Warfarin		1/16(6.3%)	0/34(0.0%)	0/8(0.0%)	1/13(7.7%)	2/71(2.8%)
Heparin		1/16(6.3%)	1/34(2.9%)	0/8(0.0%)	0/13(0.0%)	2/71(2.8%)
Beta blockers		3/16(18.8%)	6/34(17.6%)	0/8(0.0%)	3/13(23.1%)	12/71(16.9%)
Calcium channel antagonists		5/16(31.3%)	2/34(5.9%)	0/8(0.0%)	0/13(0.0%)	7/71(9.9%)
Nitrates		1/16(6.3%)	1/34(2.9%)	1/8(12.5%)	0/13(0.0%)	3/71(4.2%)
Potassium channel activators		1/16(6.3%)	0/34(0.0%)	0/8(0.0%)	1/13(7.7%)	2/71(2.8%)
Lipid lowering agent		12/16(75.0%)	4/34(11.8%)	2/8(25.0%)	4/13(30.8%)	22/71(31.0%)
ACE inhibitors		4/16(25.0%)	3/34(8.8%)	2/8(25.0%)	4/13(30.8%)	13/71(18.3%)
Angiotensin-II antagonists		4/16(25.0%)	1/34(2.9%)	1/8(12.5%)	0/13(0.0%)	6/71(8.5%)
Thiazide diuretics		2/16(12.5%)	2/34(5.9%)	1/8(12.5%)	0/13(0.0%)	5/71(7.0%)
Digoxin		1/16(6.3%)	0/34(0.0%)	0/8(0.0%)	0/13(0.0%)	1/71(1.4%)
Metformin		8/16(50.0%)	0/34(0.0%)	2/8(25.0%)	0/13(0.0%)	10/71(14.1%)
Sulfonylureas		1/16(6.3%)	0/34(0.0%)	0/8(0.0%)	0/13(0.0%)	1/71(1.4%)
Insulin		9/16(56.3%)	0/34(0.0%)	1/8(12.5%)	0/13(0.0%)	10/71(14.1%)
Other medication		9/16(56.3%)	16/34(47.1%)	6/8(75.0%)	5/13(38.5%)	36/71(50.7%)

Data are presented as median (IQR), mean (SD), or *n* (%).

Missing data (STEMI with DM, STEMI without DM, NSTEMI with DM, NSTEMI without DM):

^a^Data missing for 3 patients (0, 3, 0, 0).

DM, diabetes mellitus, STEMI, ST-elevation myocardial infarction; NSTEMI, Non-ST-elevation myocardial infarction; IQR, interquartile range; BMI, body mass index; SD, standard deviation; ACE, angiotensin-converting-enzyme; NYHA, New York Heart Association; CCS, Canadian Cardiovascular Society; MI, myocardial infarction; PCI, percutaneous coronary intervention; CABG, coronary artery bypass graft.

### Operative details and perioperative laboratory measurements

As shown in Table 2, PCI was performed in 64/71 (90.1%) participants. The average procedure time was 45 min (IQR 38 to 65.5 min). Aspirin was given pre-procedure in all participants and clopidogrel was given in under 50% of participants. Approximately 50% of participants had bystander disease in one or more vessels. Perioperative laboratory measurements on day 0 are given in Supplementary Table 4.

**TABLE 2:** Operative details.

Operative detail		STEMI with DM (*n* = 16)	STEMI without DM (*n* = 34)	NSTEMI with DM (*n* = 8)	NSTEMI without DM (*n* = 13)	Overall (*n* = 71)
PCI procedure performed		15/16(93.8%)	34/34(100.0%)	6/8(75.0%)	9/13(69.2%)	64/71(90.1%)
Total procedure time (min)		39(30.0,44.0)	45(38.0,60.0)	69(39.0,72.0)	68(65.0,75.0)	45(38.0,65.5)
Total contrast (ml)		170(100.0,220.0)	200(165.0,260.0)	225(220.0,230.0)	260(220.0,330.0)	220(160.0,260.0)
Aspirin given pre-procedure		15/15(100.0%)	34/34(100.0%)	6/6(100.0%)	9/9(100.0%)	64/64(100.0%)
Clopidogrel given pre-procedure		4/15(26.7%)	12/34(35.3%)	6/6(100.0%)	8/9(88.9%)	30/64(46.9%)
Prasugrel given pre-procedure		11/12(91.7%)	22/23(95.7%)	0/3(0.0%)	1/7(14.3%)	34/45(75.6%)
Glycoprotein used		4/15(26.7%)	11/34(32.4%)	3/6(50.0%)	3/9(33.3%)	21/64(32.8%)
If yes, type	Reopro	3/4(75.0%)	10/11(90.9%)	1/3(33.3%)	2/3(66.7%)	16/21(76.2%)
	Tirofiban	1/4(25.0%)	1/11(9.1%)	2/3(66.7%)	1/3(33.3%)	5/21(23.8%)
Number of guide catheters used	0	0/15(0.0%)	0/34(0.0%)	1/6(16.7%)	0/9(0.0%)	1/64(1.6%)
	1	15/15(100.0%)	30/34(88.2%)	3/6(50.0%)	9/9(100.0%)	57/64(89.1%)
	2	0/15(0.0%)	2/34(5.9%)	2/6(33.3%)	0/9(0.0%)	4/64(6.3%)
	3	0/15(0.0%)	2/34(5.9%)	0/6(0.0%)	0/9(0.0%)	2/64(3.1%)
Number of guide wires used	0	0/15(0.0%)	0/34(0.0%)	1/6(16.7%)	0/9(0.0%)	1/64(1.6%)
	1	14/15(93.3%)	27/34(79.4%)	2/6(33.3%)	7/9(77.8%)	50/64(78.1%)
	2	1/15(6.7%)	4/34(11.8%)	2/6(33.3%)	2/9(22.2%)	9/64(14.1%)
	3	0/15(0.0%)	3/34(8.8%)	1/6(16.7%)	0/9(0.0%)	4/64(6.3%)
Number of balloons used	0	3/15(20.0%)	6/34(17.6%)	1/6(16.7%)	0/9(0.0%)	10/64(15.6%)
	1	9/15(60.0%)	16/34(47.1%)	3/6(50.0%)	4/9(44.4%)	32/64(50.0%)
	2	2/15(13.3%)	9/34(26.5%)	0/6(0.0%)	5/9(55.6%)	16/64(25.0%)
	3	1/15(6.7%)	1/34(2.9%)	0/6(0.0%)	0/9(0.0%)	2/64(3.1%)
	4	0/15(0.0%)	0/34(0.0%)	2/6(33.3%)	0/9(0.0%)	2/64(3.1%)
	5	0/15(0.0%)	1/34(2.9%)	0/6(0.0%)	0/9(0.0%)	1/64(1.6%)
	6	0/15(0.0%)	1/34(2.9%)	0/6(0.0%)	0/9(0.0%)	1/64(1.6%)
**Target lesions**						
Target lesion	RCA	8/15(53.3%)	17/34(50.0%)	1/5(20.0%)	3/9(33.3%)	29/63(46.0%)
	LAD	4/15(26.7%)	9/34(26.5%)	4/5(80.0%)	2/9(22.2%)	19/63(30.2%)
	LCX	3/15(20.0%)	8/34(23.5%)	0/5(0.0%)	4/9(44.4%)	15/63(23.8%)
Stenosis in target lesion	>50 and ≤75	0/15(0.0%)	1/34(2.9%)	0/5(0.0%)	1/9(11.1%)	2/63(3.2%)
	>75	6/15(40.0%)	13/34(38.2%)	1/5(20.0%)	3/9(33.3%)	23/63(36.5%)
	Total coronary occlusion	9/15(60.0%)	20/34(58.8%)	4/5(80.0%)	5/9(55.6%)	38/63(60.3%)
TIMI flow in target lesion	No perfusion	8/15(53.3%)	19/34(55.9%)	4/5(80.0%)	5/9(55.6%)	36/63(57.1%)
	Dye penetration but minimal perfusion	0/15(0.0%)	3/34(8.8%)	0/5(0.0%)	0/9(0.0%)	3/63(4.8%)
	Reduced perfusion	2/15(13.3%)	9/34(26.5%)	0/5(0.0%)	0/9(0.0%)	11/63(17.5%)
	Normal perfusion	5/15(33.3%)	3/34(8.8%)	1/5(20.0%)	4/9(44.4%)	13/63(20.6%)
Bystander disease	No additional vessels	7/15(46.7%)	16/34(47.1%)	2/5(40.0%)	6/9(66.7%)	31/63(49.2%)
	1 vessel	7/15(46.7%)	12/34(35.3%)	2/5(40.0%)	3/9(33.3%)	24/63(38.1%)
	2 vessels	1/15(6.7%)	6/34(17.6%)	1/5(20.0%)	0/9(0.0%)	8/63(12.7%)
**Lesion 1:**						
TIMI flow pre-procedure	No perfusion	9/15(60.0%)	20/34(58.8%)	4/5(80.0%)	5/9(55.6%)	38/63(60.3%)
	Dye penetration but minimal perfusion	1/15(6.7%)	3/34(8.8%)	0/5(0.0%)	0/9(0.0%)	4/63(6.3%)
	Reduced perfusion	1/15(6.7%)	8/34(23.5%)	0/5(0.0%)	0/9(0.0%)	9/63(14.3%)
	Normal perfusion	4/15(26.7%)	3/34(8.8%)	1/5(20.0%)	4/9(44.4%)	12/63(19.0%)
Stent implanted		14/15(93.3%)	31/34(91.2%)	3/5(60.0%)	9/9(100.0%)	57/63(90.5%)
Stent length (mm)		19(18.0,23.0)	23(18.0,28.0)	18(16.0,28.0)	23(18.0,24.0)	23(18.0,24.0)
Stent diameter (mm)		3(3.0,3.5)	3(2.8,3.5)	3(2.5,3.0)	3(2.5,3.5)	3(2.8,3.5)
TIMI flow post-procedure	No perfusion	0/15(0.0%)	0/34(0.0%)	1/5(20.0%)	0/9(0.0%)	1/63(1.6%)
	Dye penetration but minimal perfusion	0/15(0.0%)	1/34(2.9%)	1/5(20.0%)	0/9(0.0%)	2/63(3.2%)
	Reduced perfusion	1/15(6.7%)	1/34(2.9%)	0/5(0.0%)	1/9(11.1%)	3/63(4.8%)
	Normal perfusion	14/15(93.3%)	32/34(94.1%)	3/5(60.0%)	8/9(88.9%)	57/63(90.5%)
PCI result	Good	15/15(100.0%)	32/34(94.1%)	2/5(40.0%)	8/9(88.9%)	57/63(90.5%)
	Failure	0/15(0.0%)	0/34(0.0%)	1/5(20.0%)	0/9(0.0%)	1/63(1.6%)
	Suboptimal	0/15(0.0%)	2/34(5.9%)	2/5(40.0%)	1/9(11.1%)	5/63(7.9%)
**Lesion 2:**						
TIMI flow pre-procedure	No perfusion	0/1(0.0%)	4/10(40.0%)	1/2(50.0%)	0/3(0.0%)	5/16(31.3%)
	Dye penetration but minimal perfusion	0/1(0.0%)	3/10(30.0%)	0/2(0.0%)	0/3(0.0%)	3/16(18.8%)
	Reduced perfusion	0/1(0.0%)	2/10(20.0%)	0/2(0.0%)	0/3(0.0%)	2/16(12.5%)
	Normal perfusion	1/1(100.0%)	1/10(10.0%)	1/2(50.0%)	3/3(100.0%)	6/16(37.5%)
Stent implanted		1/1(100.0%)	10/10(100.0%)	1/2(50.0%)	3/3(100.0%)	15/16(93.8%)
Stent length (mm)		28(28.0,28.0)	18(16.0,23.0)	23(23.0,23.0)	24(18.0,38.0)	20(16.0,28.0)
Stent diameter (mm)		3(2.8,2.8)	3(3.0,3.5)	3(3.0,3.0)	4(2.8,7.3)	3(3.0,3.5)
TIMI flow post-procedure	Normal perfusion	1/1(100.0%)	10/10(100.0%)	2/2(100.0%)	3/3(100.0%)	16/16(100.0%)
PCI result	Good	1/1(100.0%)	10/10(100.0%)	1/2(50.0%)	3/3(100.0%)	15/16(93.8%)
	Suboptimal	0/1(0.0%)	0/10(0.0%)	1/2(50.0%)	0/3(0.0%)	1/16(6.3%)
**Lesion 3:**						
TIMI flow pre-procedure	Dye penetration but minimal perfusion		1/2(50.0%)		0/1(0.0%)	1/3(33.3%)
	Normal perfusion		1/2(50.0%)		1/1(100.0%)	2/3(66.7%)
Stent implanted			1/2(50.0%)		1/1(100.0%)	2/3(66.7%)
Stent length (mm)			20(20.0,20.0)		38(38.0,38.0)	29(20.0,38.0)
Stent diameter (mm)			3(3.0,3.0)		3(2.5,2.5)	3(2.5,3.0)
TIMI flow post-procedure	Dye penetration but minimal perfusion		1/2(50.0%)		0/1(0.0%)	1/3(33.3%)
	Normal perfusion		1/2(50.0%)		1/1(100.0%)	2/3(66.7%)
PCI result	Good		1/2(50.0%)		1/1(100.0%)	2/3(66.7%)
	Not treated		1/2(50.0%)		0/1(0.0%)	1/3(33.3%)
**Lesion 4:**						
TIMI flow pre-procedure	Normal perfusion		1/1(100.0%)		1/1(100.0%)	2/2(100.0%)
Stent implanted			1/1(100.0%)		1/1(100.0%)	2/2(100.0%)
Stent length (mm)			16(16.0,16.0)		16(16.0,16.0)	16(16.0,16.0)
Stent diameter (mm)			3(3.0,3.0)		3(3.0,3.0)	3(3.0,3.0)
TIMI flow post-procedure	Normal perfusion		1/1(100.0%)		1/1(100.0%)	2/2(100.0%)
PCI result	Good		1/1(100.0%)		1/1(100.0%)	2/2(100.0%)

Data are presented as *n* (%).

DM, diabetes mellitus; STEMI, ST-elevation myocardial infarction; NSTEMI, Non-ST-elevation myocardial infarction; TIMI, thrombolysis in myocardial infarction; PCI, percutaneous coronary intervention; RCA, right coronary artery; LAD, left anterior descending; LCX, left circumflex.

### Medications

Medications given at hospital discharge are reported in Supplementary Table 5. All patients were prescribed aspirin and a lipid-lowering agent at discharge.

### Cardiac magnetic resonance imaging assessments and circulating progenitor cell measurements

Sixty of the 71 participants had a cardiac MRI at baseline (14 STEMI with DM, 29 STEMI without DM, 6 NSTEMI with DM, 11 NSTEMI without DM), and 55 had a cardiac MRI at three months (11 STEMI with DM, 27 STEMI without DM, 7 NSTEMI with DM, 10 NSTEMI without DM). Of the latter 55, 52 had both baseline and 3-month MRI (11 STEMI with DM, 26 STEMI without DM, 5 NSTEMI with DM, 10 NSTEMI without DM). The number of patients with CPC laboratory and MRI data available is given in Supplementary Table 6.

### Objective 1: Proportion and migratory capacity of circulating progenitor cells

**FIGURE 2:**
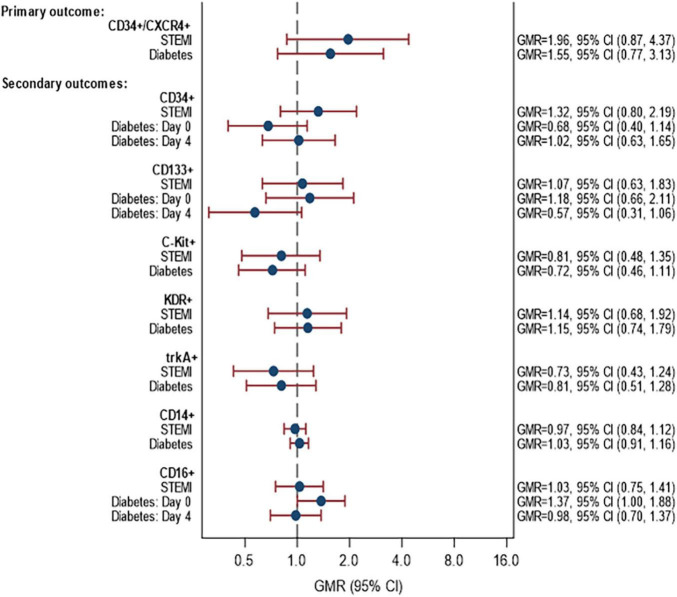
Associations between STEMI and DM with proportion of CPCs. Models fitted to participants with lab data available on day 0 and/or day 4: 58 participants (14 STEMI with DM, 31 STEMI without DM, 6 NSTEMI with DM, 7 NSTEMI without DM). Estimates are adjusted for age, sex and time since onset of symptoms. *p*-Values for interaction STEMI × diabetes: CD34+/CXCR4+ (*p* = 0.2967), CD34+ (*p* = 0.3257), CD133+ (*p* = 0.1023), C-Kit (*p* = 0.8526), KDR+ (*p* = 0.6713), trkA+ (*p* = 0.1884), CD14+ (*p* = 0.9978), CD16+ (*p* = 0.7661). *p*-Values for interaction STEMI × time: CD34+/CXCR4+ (*p* = 0.7517), CD34+ (*p* = 0.1756), CD133+ (*p* = 0.9222), C-Kit (*p* = 0.5820), KDR+ (*p* = 0.1839), trkA+ (*p* = 0.7548), CD14+ (*p* = 0.4178), CD16+ (*p* = 0.1561). *p*-Values for interaction diabetes × time: CD34+/CXCR4+ (*p* = 0.8349), CD34+ (*p* = 0.095), CD133+ (*p* = 0.0649), C-Kit (*p* = 0.660), KDR+ (*p* = 0.4274), trkA+ (*p* = 0.8302), CD14+ (*p* = 0.5026), CD16+ (*p* = 0.0551) STEMI, ST-elevation myocardial infarction; NSTEMI, non-ST-elevation myocardial infarction.

Data on the abundance of CPCs in PB at day 0 and day 4 days post-MI is shown in Supplementary Table 7. The associations between STEMI and DM and the percentage of CPCs for the various cell markers are given in Figure 2. In summary, there were no statistically significant differences in the rate of CPCs between patients with/without STEMI and with/without DM for any of the cell markers. For the primary outcome, the proportion of CD34+/CXCR4+ cells was on average 1.96 times higher in STEMI than NSTEMI (GMR = 1.96, 95% CI 0.87–4.37). This difference was not statistically significant. Likewise, the proportion of CD34+/CXCR4+ cells tended to be higher in patients with DM compared to patients without DM (GMR = 1.55, 95% CI 0.77, 3.13). There was no evidence of an interaction between STEMI ± and DM (*p* = 0.30).

When looking at the change in the percentage of CD34+/CXCR4+ cells from day 0 to day 4, the results show no association between STEMI or DM and change in CD34/CXCR4 cells (Supplementary Figure 2).

**FIGURE 3:**
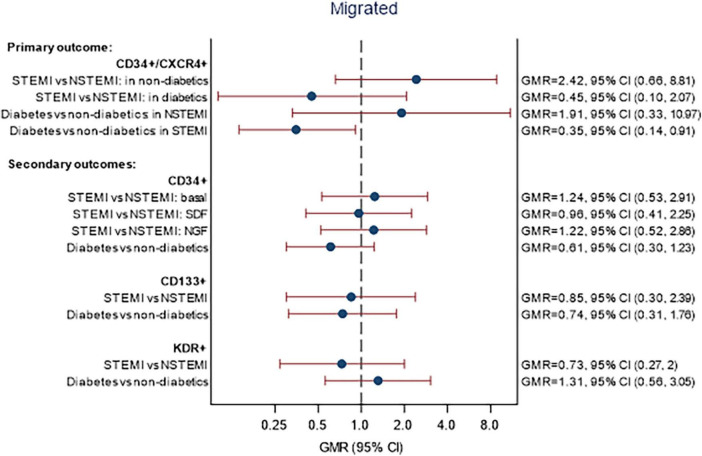
Associations between STEMI and DM with migration of CPCs. Models fitted to participants with migratory lab data available on day 4: 52 participants (14 STEMI with DM, 27 STEMI without DM, 5 NSTEMI with DM, 6 NSTEMI without DM). Estimates are adjusted for age, sex, and time since onset of symptoms. Migrated—*p*-values for interaction STEMI × DM: CD34+/CXCR4+ (*p* = 0.0983), CD34+ (*p* = 0.3235), CD133+ (*p* = 0.7571), KDR+ (*p* = 0.1383). *p*-Values for interaction STEMI × chemo-attractant: CD34+/CXCR4+ (*p* = 0.1217), CD34+ (*p* = 0.0908), CD133+ (*p* = 0.9977), KDR+ (*p* = 0.5262).

A summary of CPC migration data is shown in Supplementary Table 8. The associations between STEMI and DM and the percentage of migrated cell markers on day 4 are given in Figure 3. There were no statistically significant differences in the proportion of migrated CPCs between patients with STEMI vs NSTEMI and DM vs non-DM for any of the cell markers. The relationship between STEMI/NSTEMI and CD34+/CXCR4+ cells was different in patients with DM than in those without DM. In patients without DM, STEMI was associated with over double the proportion of migrated CD34+/CXCR4+ cells compared to NSTEMI (GMR 2.42, 95% CI 0.66, 8.81). In patients with DM, the association was in the opposite direction, with a 55% reduction in migrated CD34+/CXCR4+ cells (GMR = 0.45, 95% CI 0.10, 2.07). However, it is essential to note that there are small numbers of patients within the subgroups (see footnote to Figure 3 for numbers) and, although the interaction was included (*p* = 0.098), the association of STEMI/NSTEMI with migrated CD34+/CXCR4+ cells was not statistically significant in either group.

Finally, there was no significant association between peak troponin T or CRP on day 0 or nearest and the percentage of CPCs for the various cell markers (Supplementary Figure 3).

### Objective 2: Magnetic resonance imaging and clinical outcomes

**FIGURE 4:**
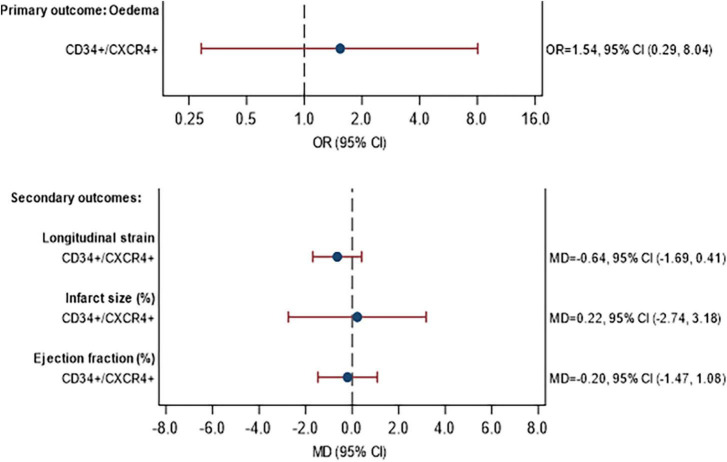
Association between CD34+/CXCR4+ frequency and primary and secondary MRI outcomes at 3 months. Model fitted to participants with MRI and CPC data availabl43 participants (11 STEMI with DM, 24 STEMI without DM, 3 NSTEMI with DM, 5 NSTEMI without DM), excluding participants with missing data (see below). Edema: excluding 2 participants with missing edema data at baseline or 3 months. Longitudinal strain: excluding 3 participants with missing Longitudinal strain data at baseline or 3 months. Infarct size: excluding 3 participants with missing infarct size data at baseline or 3 months. Ejection fraction: excluding 0 participants with missing ejection fraction data at baseline or 3 months. Estimates are adjusted for STEMI, diabetes, age, sex, time since onset of symptoms and baseline value of outcome being analyzed. CI, confidence interval; GMR, geometric mean ratio; OR, odds ratio; MD, mean difference.

**FIGURE 5:**
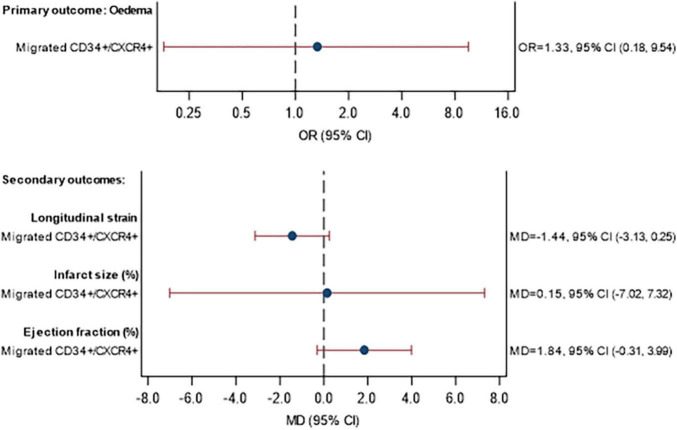
Association between CD34+/CXCR4+ migration and primary and secondary MRI outcomes at 3 months. Model fitted to participants with MRI and migrated CPC data available: 40 participants (11 STEMI with DM, 21 STEMI without DM, 3 NSTEMI with DM, 5 NSTEMI without DM), excluding participants with missing data (see below). Eedema: excluding 1 participant with missing eedema data at baseline or 3 months. Longitudinal strain: excluding 1 participant with missing Longitudinal strain data at baseline or 3 months. Infarct size: excluding 2 participants with missing infarct size data at baseline or 3 months Ejection fraction: excluding 0 participants with missing ejection fraction data at baseline or 3 months. Estimates are adjusted for STEMI, diabetes, age, sex, time since onset of symptoms and baseline value of outcome being analyzed. CI, confidence interval; GMR, geometric mean ratio; OR, odds ratio; MD, mean difference.

The MRI data are shown in Supplementary Table 9 for the assessment at baseline and in Supplementary Table 10 for the 3-month follow-up. The association between (a) the percentage of CD34+/CXCR4+ cells and (b) the percentage of migrated CD34+/CXCR4+ cells, with MRI outcomes are given in Figures 4, 5, respectively. No statistically significant associations were observed between the proportion of CD34+/CXCR4+ cells and MRI outcomes. There were also no statistically significant associations between migrated CD34+/CXCR4+ and the MRI outcomes, although effect sizes were stronger. For each unit increase in the proportion of migrated CD34+/CXCR4+, the longitudinal strain was on average 1.44 units lower (MD = −1.44, 95% CI −3.13, 0.25) and LVEF was 1.84 units higher (MD = 1.84, 95% CI −0.31, 3.99).

Clinical outcomes are summarized at each time point by STEMI and DM status in Table 3. Outcomes were not formally compared, as specified *a priori* in the study protocol. Overall, 12.7% of participants (9/71) experienced a major adverse cardiac-related event (MACE) during follow-up.

**TABLE 3:** Clinical outcomes.

		STEMI with DM (*n* = 16)	STEMI without DM (*n* = 34)	NSTEMI with DM (*n* = 8)	NSTEMI without DM (*n* = 13)	Overall (*n* = 71)
**Event**						
MACE	Any event	4/16(25.0%)	2/34(5.9%)	0/8(0.0%)	3/13(23.1%)	9/71(12.7%)
Death	Overall	0/16(0.0%)	0/34(0.0%)	0/8(0.0%)	0/13(0.0%)	0/71(0.0%)
	28 days	0/16(0.0%)	0/34(0.0%)	0/8(0.0%)	0/13(0.0%)	0/71(0.0%)
	3 months	0/16(0.0%)	0/34(0.0%)	0/8(0.0%)	0/13(0.0%)	0/71(0.0%)
	12 months	0/16(0.0%)	0/34(0.0%)	0/8(0.0%)	0/13(0.0%)	0/71(0.0%)
New myocardial infarction	Overall	2/16(12.5%)	0/34(0.0%)	0/8(0.0%)	2/13(15.4%)	4/71(5.6%)
	28 days	1/16(6.3%)	0/34(0.0%)	0/8(0.0%)	0/13(0.0%)	1/71(1.4%)
	3 months	0/16(0.0%)	0/34(0.0%)	0/8(0.0%)	0/13(0.0%)	0/71(0.0%)
	12 months	1/16(6.3%)	0/34(0.0%)	0/8(0.0%)	2/13(15.4%)	3/71(4.2%)
Further revascularisation	Overall	2/16(12.5%)	2/34(5.9%)	0/8(0.0%)	2/13(15.4%)	6/71(8.5%)
	28 days	1/16(6.3%)	1/34(2.9%)	0/8(0.0%)	0/13(0.0%)	2/71(2.8%)
	3 months	0/16(0.0%)	1/34(2.9%)	0/8(0.0%)	0/13(0.0%)	1/71(1.4%)
	12 months	1/16(6.3%)	0/34(0.0%)	0/8(0.0%)	2/13(15.4%)	3/71(4.2%)
Recurrent angina	Overall	1/16(6.3%)	0/34(0.0%)	0/8(0.0%)	0/13(0.0%)	1/71(1.4%)
	28 days	0/16(0.0%)	0/34(0.0%)	0/8(0.0%)	0/13(0.0%)	0/71(0.0%)
	3 months	0/16(0.0%)	0/34(0.0%)	0/8(0.0%)	0/13(0.0%)	0/71(0.0%)
	12 months	1/16(6.3%)	0/34(0.0%)	0/8(0.0%)	0/13(0.0%)	1/71(1.4%)
Any SAE	Overall	6/16(37.5%)	5/34(14.7%)	1/8(12.5%)	4/13(30.8%)	16/71(22.5%)
	28 days	6/16(37.5%)	5/34(14.7%)	1/8(12.5%)	4/13(30.8%)	16/71(22.5%)
	3 months	0/16(0.0%)	0/34(0.0%)	0/8(0.0%)	0/13(0.0%)	0/71(0.0%)
	12 months	0/16(0.0%)	0/34(0.0%)	0/8(0.0%)	0/13(0.0%)	0/71(0.0%)

If event not recorded (missing) it is assumed event did not occur.

DM, diabetes mellitus; STEMI, ST-elevation myocardial infarction; NSTEMI, non-ST-elevation myocardial infarction; SAE, serious adverse event; MACE, major adverse cardiac-related eve.

Supplementary Table 11 presents all adverse events (AEs) and SAEs reported during the study (in-hospital and during follow-up). Further details of unexpected SAEs are given in Supplementary Table 12.

## Discussion

Cardiac repair after MI depends on the balance between the magnitude of ischemic injury and a regulated inflammatory and wound-healing response: larger infarcts and depressed reparative mechanisms increase the risk for developing adverse ventricular remodeling and heart failure (30). Accumulating evidence indicates that several cell types are mobilized from distant niches and reach the ischemic heart guided by a chemokine gradient. The cell migratory response has been modeled using an *in vitro* assay to generate useful prognostic and therapeutic insights (17). The medical value of the approach may be implemented by studying sub-categories of patients classified according to the severity of tissue injury and associated risk factors.

The present study indicates that DM impacts the relationship between myocardial damage and progenitor cell migratory response. CPC migratory activity was higher in STEMI vs. NSTEMI. However, in patients without DM, the effect of STEMI vs NSTEMI was GMR = 2.42. In patients with DM, the effect of STEMI vs. NSTEMI was GMR = 0.45; hence, the property of STEMI vs. NSTEMI was reversed in the presence of DM. In addition, there was a trend for a positive association between CPC migratory activity and LVEF at 3 months follow-up, following adjustment for STEMI, DM, age, sex, time since onset of symptoms and baseline LVEF. This suggests that the outcome after MI is advantaged in patients who have CPCs endowed with efficient migratory activity.

Shintani et al. were the first to describe the rapid mobilization of CD34+ cells in acute MI (31). The abundance of circulating CPCs peaked 7 days after the onset of MI and paralleled the significant increase in blood levels of angiogenic factors and chemokines. Moreover, nociceptive signaling to the BM can contribute to the regulation of CPC liberation from the BM (15). Massa et al. showed that hematopoietic CPCs follow the mobilization kinetics of endothelial progenitor cells; (5) however, there are discrepancies between reports regarding the time for CPCs to reach the highest levels in the PB (6, 7). A meta-analysis by Rigato et al. showed that the numerical reduction of circulating CPCs, in particular CD34+/CD133+ hematopoietic cells, was associated with a 2-fold increased risk of future cardiovascular events and cardiovascular death in patients with suspected coronary artery disease, acute coronary syndrome, previous stroke, or in patients without acute events but with cardiovascular risk factors (32). The quantitative assessment of this cell population was capable of predicting long-term adverse cardiovascular outcomes and multi-organ damage in DM patients (19, 33).

A novel aspect investigated here is the change occurring in CPC quantity during the first few days after an MI; with the expected result being that the infarct severity would influence the number of CPCs in the peripheral blood. To this aim, we measured the relative abundance of CD34+/CXCR4 CPCs on two occasions, during the first 24 h and between 84 and 132 h after MI. Results indicate that STEMI was more potent than NSTEMI in determining an increase in circulating CPCs. Intriguingly, the relative abundance of CD34+/CXCR4 CPCs in PB tended to be higher in the PB of patients with DM independent of a STEMI or NSTEMI. This result apparently contradicts the notion of a defective mobilization, alias mobilopathy, initially reported by Di Persio in people with DM (10). The sampling schedule adopted in the present study might not have been able to capture such mobilopathy. This may also explain the difference between our data and previous reports suggesting that the CPC abundance can predict the late outcome post-MI (19). In the present study, no significant association was observed between CD34+/CXCR4+ cell abundance and MRI outcomes.

We have next investigated whether the *in vitro* migratory activity of CPCs may reflect an intrinsic defect in the repair response with higher fidelity. This possibility is supported by the observation that migration *in vitro* was (1) directly proportional to the extent of myocardial damage in the comparison between STEMI vs. NSTEMI, (2) reversed in the presence of DM, and (3) predictive of a better LVEF as assessed by cardiac MRI at the three months follow up. This observation extends our previous report about the predictive value of cell migration in patients with critical limb ischemia, where a multivariable regression analysis showed that CD34+ cell migration forecasted cardiovascular mortality during a 6-year follow-up (17). This was interpreted as the consequence of altered paracrine signaling, involving the TUG1 sponge/miRNA-21/PDCD4 axis, which conveys antiangiogenic and proapoptotic features from CD34+ cells to the endothelium (17). Further research is needed to determine whether a similar mechanism is operative in CPCs mobilized after an acute MI and influenced by DM and cardiovascular risk factors.

### Strengths and limitations

Despite attempts to reduce bias, this hypothesis-generating, observational study has limitations intrinsic to the small sample size. In particular, the recruitment reached the initial goal for STEMI but not for NSTEMI or cardiac MRI. In addition, no formal adjustment was made for multiple testing due to the hypothesis-generating nature of the study. Moreover, the migration assay is an *in vitro* test that does not reflect cell extrinsic determinants of mobilization, such as the state of the bone marrow niche, neurogenic and neurotrophic influences, and metabolic status (14, 34–40). Regardless of these limitations, the data collected for the study are, we believe, the only ones that exist regarding an interaction between infarct and diabetes on the CPC migratory activity—hence the importance of reporting the findings. The data reinforce the concept that cell migration especially if combined with an assessment of the molecular profile of migrated cells may provide useful clinical information in ischemic cardiovascular disease.

## Data availability statement

The original contributions presented in this study are included in the article/Supplementary material, further inquiries can be directed to PM, mdprm@bristol.ac.uk.

## Ethics statement

The studies involving human participants were reviewed and approved by Wiltshire Research 98 Ethics Committee. The patients/participants provided their written informed consent to participate in this study.

## Author contributions

PM was responsible as corresponding author for the final writing and the data presented. All authors conceived and designed the article and read and approved the final manuscript.
