# Supplementary material for: A cohort study of circulating progenitor cells after ST-segment elevation and non-ST segment elevation myocardial infarction in non-diabetic and diabetic patients
Source: Front Cardiovasc Med. 2022 Nov 17;9:1011140. doi: 10.3389/fcvm.2022.1011140 (PMC9714619; doi:10.3389/fcvm.2022.1011140)
Supplement: Supplementary file 1 [file Data_Sheet_1.PDF]

Supplementary Appendix

Contents

ProMIS Study protocol........ 2
Supplementary tables and figures........ 22

Table of Supplementary Figures

Supplementary Figure 1: Schema of the study 22
Supplementary Figure 2: Post-hoc analysis - associations between STEMI and diabetes with respect to percentage of CD34+/CXCR4+ on day 4 adjusting for CD34+/CXCR4+ on day 0........ 23
Supplementary Figure 3: Associations between Troponin T and CRP and the percentage of CPCs........ 24

Table of Supplementary Tables

Supplementary Table 1: Time points of data collection........ 25
Supplementary Table 2: Details of withdrawals........ 26
Supplementary Table 3: Protocol deviations 27
Supplementary Table 4: Perioperative lab measurements on day 0* 28
Supplementary Table 5: Medications at hospital discharge and during follow-up........ 30
Supplementary Table 6: CPC lab data and MRI data available 34
Supplementary Table 7: CPC measurements 35
Supplementary Table 8: Migrated cells on day 4 37
Supplementary Table 9: Cardiac MRI at baseline........ 38
Supplementary Table 10: Cardiac MRI at 3 months........ 39
Supplementary Table 11: Expected adverse events and serious adverse events........ 40
Supplementary Table 12: Details of unexpected serious adverse events........ 43

Appendix 1 - ProMIS Study Protocol
Version 9.0 dated 30th February 2013

Short project title: Progenitor cell response after **M**yocardial **I**nfarction **S**tudy (ProMIS)

Project Title: A cohort study of circulating progenitor cells after ST segment elevation and non-ST segment elevation myocardial infarction in non-diabetic and diabetic patients.

REC reference number:	09/H0104/58
R&D reference number:	CS 2009/3297
Funding reference:	2008/SS/BRU
ISRCTN:	19569306

Details of sponsor
University Hospitals Bristol NHS Foundation Trust
Research and Innovation Department
Level 3 – Education Centre
Upper Maudlin Street
Bristol BS2 8AE
Tel: 0117 342 0233
Fax: 0117 342 0239

Chief Investigators & Research Team Contact Details

Dr Andreas Baumbach
Consultant Cardiologist
Honorary Reader in Cardiology
University Hospitals Bristol NHS Foundation Trust
Bristol Heart Institute
Tel: 0117 342 6631
E-mail: andreas.baumbach@uhbristol.nhs.uk

Dr Tom Johnson
Consultant Cardiologist
University Hospitals Bristol NHS Foundation Trust
Bristol Heart Institute
Tel: 0117 342 6573
E-mail: tom.johnson@uhbristol.nhs.uk

Dr Costanza Emanueli
Professorial Research Fellow in Vascular Pathology & Regeneration
University of Bristol
Bristol Royal Infirmary, Level 7
Marlborough Street
Bristol BS2 8HW
Tel: 0117 342 3512
E-mail: costanza.emanueli@bristol.ac.uk

Professor Paolo Madeddu
Professor of Experimental Cardiovascular Medicine
University of Bristol
Bristol Royal Infirmary, Level 7
Marlborough Street
Bristol BS2 8HW
Tel: 0117 342 3904
E-mail: mdprm@bristol.ac.uk

Dr Chris Rogers
Senior Research Fellow in Medical Statistics,
Clinical Trials and Evaluation Unit
University of Bristol
Bristol Royal Infirmary, Level 7
Marlborough Street
Bristol BS2 8HW
Tel: 0117 342 2507
Fax: 0117 342 3288
Email: chris.rogers@bris.ac.uk

Professor Barnaby Reeves
Professorial Research Fellow in Health Services Research
Clinical Trials and Evaluation Unit
University of Bristol
Bristol Royal Infirmary, Level 7
Marlborough Street
Bristol BS2 8HW
Tel: 0117 342 3143
Fax: 0117 342 3288
Email: barney.reeves@bristol.ac.uk

Dr Chiara Bucciarelli-Ducci
Senior Lecturer in Non-Invasive Cardiac Imaging
University Hospitals Bristol NHS Foundation Trust
Bristol Heart Institute
Bristol Royal Infirmary, Level 7
Malborough Street
Bristol BS2 8HW
Email: c.bucciarelli-ducci@rbht.nhs.uk

Dr Mark Hamilton
Consultant Cardiac Radiologist
Bristol Royal Infirmary
Queens Building Level 3
Marlborough Street
Bristol BS2 8HW
Tel: 0117 342 2729
Fax: 0117 928 3267
Email: mark.hamilton@uhbristol.nhs.uk

1 Glossary

BMC	Bone Marrow Cells
CPC	Circulating Progenitor Cells
CTEU	Clinical Trials and Evaluation Unit
CRF	Case Report Form
DM	Diabetes Mellitus
DMSC	Data Monitoring and Safety Committee
ECG	Electrocardiogram
EF	Ejection Fraction
EPC	Endothelial Progenitor Cells
ICH GCP	International Conference for Harmonisation of Good Clinical Practice
MRI	Magnetic Resonance Imaging
NIHR	National Institute for Health Research
NSTEMI	Non ST segment Elevation Myocardial Infarction
PCI	Percutaneous Intervention
PIS	Participant Information Sheet
RCT	Randomised Controlled Trial
SAE	Serious Adverse Event
STEMI	ST segment Elevation Myocardial Infarction
SMG	Study Management Group
SSG	Study Steering Group
UHBristol	University Hospitals Bristol NHS Foundation Trust

2 Definitions

DM	Participants with an established diagnosis of Diabetes Mellitus
MI	Myocardial infarction occurs when the blood supply to part of the heart is interrupted causing some heart cells to die; death of cells can be established by standard diagnostic criteria (symptoms, ECG changes, release of cardiac enzymes, imaging investigations).
NSTEMI	Defined by the evidence of myocardial necrosis (troponin elevation), with or without ECG changes indicative of ischaemia (including ST depression and t wave inversion but excluding an ST elevation as described for STEMI).
STEMI	Determined by an ECG at the time of the MI, an ST elevation myocardial infarction is associated with either an ST elevation in 2 contiguous leads of a 12-lead ECG, or new onset left bundle branch block.

3 Lay summary

In this study we are trying to find out how some natural repair mechanisms react after a heart attack and whether diabetes interferes with these natural responses, thereby worsening the patient's clinical outcome. We know that, following a heart attack, the body increases the production of stem cells that are designed to help new blood vessels to grow and thereby repair the damaged heart. We want to find out whether the size of the response is influenced by the

amount of damage to the heart and, if so, whether this relationship between the response and amount of damage is lost in patients with diabetes. Counting these cells in a blood sample and measuring how well they respond to chemical stimuli produced by damaged heart muscle may help us to identify which patients need more aggressive treatment after a heart attack.

One objective of this study is to improve our understanding of how the number and behaviour of stem cells in the blood vary over time after a heart attack. We will do this by measuring the number of stem cells in a blood sample collected after a heart attack and assessing the ability of the cells to move towards a chemical stimulus in a test tube. A second objective is to explore whether the number of stem cells released after a heart attack influences the extent to which the heart muscle repairs itself over a period of three months after the heart attack. A third objective is to investigate whether the number of stem cells and their ability to respond to chemicals released by damaged heart muscle differ between patients who have had mild or severe heart attacks. Some researchers think that patients with diabetes are less able to increase their production of stem cells in the blood. Therefore, a final objective is to compare the number and response of stem cells in patients with and without diabetes.

4 Background

In the last 10 years, the fundamental role of bone marrow-derived progenitor cells with a pro-angiogenic capacity in post-ischaemic healing has been firmly established.[1-3] Pro-angiogenic progenitor cells are characterised by markers of immature stem cells (CD133, CD34). In addition, these cells express receptors, like the receptor for the chemokine SDF-1, e.g. CXCR4, which guide these cells in their homing into damaged peripheral tissue. Under normal conditions, these progenitor cells represent a minor fraction of mononuclear cells (MNCs) and their function seemingly consists in ‘scouting’ in the peripheral circulation to repair minor damage to the vascular endothelium.

After an ischaemic event, such as a myocardial infarction (MI), however, leukocytes and pro-angiogenic progenitor cells are mobilised in abundance from bone marrow into the circulation, becoming circulating progenitor cells (CPCs). The proportion of CPCs compared to other MNC fractions increases rapidly during the first days after an MI, then slowly returns to baseline levels. CPCs are recruited from the circulation to the ischaemic heart attracted by chemical signals released at the infarct site. Once CPCs reach the damaged tissue, they promote the formation of new arterial vessels and possibly contribute to cardiac regeneration. CPCs could directly participate in these regenerative processes due to their capacity to differentiate into vascular cells and cardiomyocytes but they principally act by supporting regeneration afforded by local cells of the heart.[2,4,5]

During the process of mobilisation from bone marrow, CPCs retain some typical markers of haematopoietic stem cells, like $CD34^+$, as well as endothelial cell markers, like vascular endothelial growth factor receptor 2 (VEGFR2/KDR). As indicated above, CPCs also express various receptors instrumental to their migratory capacity. The ability of CPCs to reach the right target could be compromised if the receptors responsible for guiding migration diminish.

The incidence of diabetes mellitus (DM) is rising and represents one of the greatest medical and socioeconomic challenges worldwide. Cardiovascular events are the leading cause of mortality in DM patients and these have a worse outcome after an MI. Mechanisms underpinning poorer prognosis are the objective of intensive investigation with the hope of finding new effective therapies.

We and others have shown that DM impairs the abundance, survival and functional capacities of CPCs jeopardising the neovascularisation of the infarcted heart.[6,7] In addition, we have

shown that a moderate increase in glucose impairs the active migration and viability of human CPCs. Furthermore, pilot data suggests that in patients with DM the control of CPC mobilisation from bone marrow into the systemic circulation is impaired. The mobilisation of CPCs is associated with preferential release of pro-inflammatory cells. Therefore, a process aimed at regenerating damaged tissues may be converted by DM into an adverse phenomenon perpetuating local inflammation and impairing of cardiac function.

5 Aims and objectives

The aim of the study is to characterise the ***number*** and ***migratory capacity*** of CPCs in patients with or without DM who have had either a ST segment elevation myocardial infarction (STEMI) or a non-ST segment elevation myocardial infarction (NSTEMI).

Specific objectives of the study are:

1. To measure the ***number*** and ***migratory capacity*** of CPCs and then to test the hypotheses that;

    a. the ***number*** of CPCs differs after STEMI compared to NSTEMI;
    b. the ***migratory capacity*** of CPCs differs in patients with or without DM;
    c. the ***number*** and ***migratory capacity*** of CPCs are associated with covariates characterising the severity of the initial STEMI or NSTEMI (e.g. troponin I, hsCRP) or the quality of glucose control ($HbA1_c$).

2. To test the hypothesis that the ***number*** and ***migratory capacity*** of CPCs after a STEMI or NSTEMI influence the size of the myocardial scar and myocardial contractility three months after the initial cardiac event.

6 Plan of Investigation

6.1 Study Schema

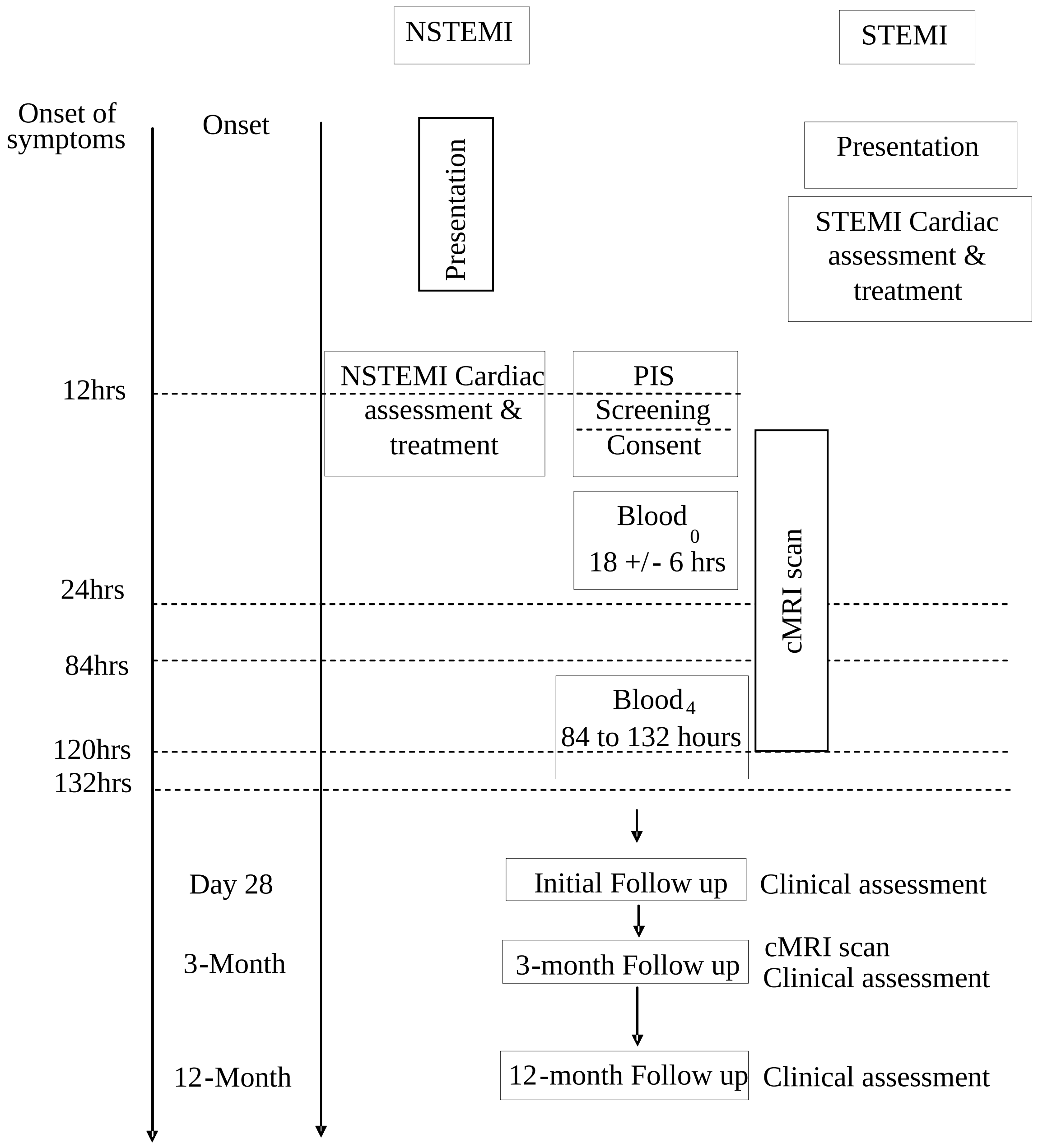

6.2 Study design

This is a longitudinal study of four cohorts, i.e. patients with and without DM who have had a STEMI or NSTEMI. We plan to recruit study participants until we have reached our target quota in all four cohorts, with a maximum of 96 participants.

6.3 Exposures

6.3.1 Exposures of primary interest

The exposures of primary interest vary depending on the objective (Section 3). Objective 1 describes the following exposures:

1(a) participants presenting with a STEMI or NSTEMI;
1(b) and (c) participants having DM or not;

For objective 2, the extent of the CPC responses in patients is the exposure of primary interest.

6.3.2 Exposures potentially underlying dichotomies

For objective 1 (d), covariates include: serum troponin I, HbA1c, blood glucose, hsCRP, symptom onset→open artery time, etc.

6.3.3 Confounding factors

We will not include any confounding factors within the primary or secondary analyses.

6.4 Outcomes

6.4.1 Primary outcomes

The primary outcome for objective 1 is the number of CPCs measured in a peripheral blood sample or the migratory ability of CPCs expressing CXCR4 to the chemo-attractant SDF-1 (assessed in a test tube by a migration assay). The primary outcome for objective 2 is the size of myocardial scar (volume or mass of affected myocardium) three months after symptom onset.

6.4.2 Secondary outcomes

Outcomes vary depending on the objective (see Section 5).

For objective 1, the secondary outcomes are:

- Number of CPCs expressing cell surface markers: CD34, CD133, c-kit, KDR, trkA, beta-2, CD14 and CD16, either viable, apoptotic or necrotic. Migratory ability of PBMNC expressing CPC surface markers: CD34, CD133, c-kit, KDR, trkA, beta-2, CD164, CD14, CD16. For migration assays, we will use SDF-1 and NGF as chemo-attractants and PBS as vehicle control
- Viability of CPCs on Day 4 for CPCs expressing CXCR4 and sub-populations of CPCs expressing cell surface markers: CD34, CD133, c-kit, KDR, trkA, beta-2, CD164, CD14 and CD16)

For objective 2, the secondary outcomes are:

- Myocardial contractility / wall thickening three months after the index STEMI or NSTEMI
- LV wall motion

The following clinical outcomes will be evaluated at Day 4, 3 and 12 months after the index admission:

- Incidence of periprocedural myocardial damage assessed by analysis of creatinine kinase (Day 4 only)
- Major adverse cardiac-related events (death, new MI, further revascularisation, recurrent angina as defined by repeat coronary angiogram for chest pain symptoms)
- Hospitalisation rates

6.5 Study population

The Bristol Royal Infirmary regularly admits patients with troponin-positive acute coronary syndromes (acute myocardial infarction (MI) or with unstable angina). These patients are treated in the Department of Cardiology. About 500 to 600 patients admitted with STEMI have primary percutaneous intervention (PCI) per year. Patients with DM account for about 20% of both STEMI and NSTEMI groups (8).

We plan to screen patients who present at the Bristol Royal Infirmary with a STEMI or NSTEMI from 1 December 2009 until we have successfully filled recruitment to all four cohorts (maximum of 96 participants). It may not be possible to recruit consecutive patients, due to the limited resources available. However, recruitment will not be influenced by the characteristics of presenting patients (other than eligibility criteria and the availability of clinical and laboratory staff to obtain and process samples, see below) and we anticipate that the study sample will be representative of the study population.

6.5.1 Inclusion criteria

A participant may enter the study if ALL of the following apply:

1. Presentation to a Bristol Heart Institute cardiologist within 24 hours after the onset of symptoms
2. Admission with STEMI or NSTEMI (troponin positive acute coronary syndromes)
3. Aged 40 to 75 at admission
4. Willing to participate for the duration of the study as defined in the study protocol

6.5.2 Exclusion criteria

A participant may not enter the study if ANY of the following apply:

1. Anaemia, i.e. haemoglobin <10mg/dl
2. Cardiogenic shock on presentation
3. Renal impairment (eGfR <50ml)
4. Haemodynamic instability
5. Contraindications to having the MRI scan (e.g. metallic implant, pacemakers, screws, claustrophobia, etc)
6. Previous coronary event within the last 12 weeks
7. Participation in another clinical study
8. Patients who are unable or unwilling to return for follow-up in accordance with the study schedule after three months
9. Heightened anxiety during recruitment

6.6 Sample size calculation

Our target is to recruit between 80 and 96 participants; patients with DM make up only a minority (about 15-20%) of patients presenting with STEMI/NSTEMI and it is challenging to recruit patients presenting with NSTEMI (delay between onset of symptoms and presentation), although diabetic patients with NSTEMI seem to present promptly. Therefore, this total will consist of at least 32 in the STEMI without DM cohort, 16 in the NSTEMI without DM cohort, 16 in the DM STEMI cohort and 16 in the DM NSTEMI cohort. Recruitment will be targeted to achieve these totals, but in order to maintain blinding of the research team (excluding those responsible for recruitment) we will continue to recruit to all cohorts up to a maximum of 96 patients (stopping before this if the target for each cohort is met).

The sample size calculations are based on the assumption that the target numbers will be met for each cohort (80 in total) and that attrition will be no more than 10%.

Objective 1(a) proposes to compare STEMI participants with NSTEMI participants, irrespective of DM status. Objective 1(b) proposes to compare participants with and without DM, irrespective of STEMI/NSTEMI classification. Assumptions underpinning the sample size calculation are that:

- the influences of the type of myocardial infarction (STEMI vs. NSTEMI) and DM are independent (i.e. Interactions between STEMI vs. NSTEMI, and participants with and without DM, are not expected)
- a baseline measure (day 0) is available and that the correlation between baseline and day 4 (defined at 84 to 132 hours) measures is 0.5
- the ratio of the numbers of STEMI: NSTEMI and without DM:DM patients is 3:2

With at least 32/16 participants per cohort as described above, both comparisons will have 80% power at a significance level of 5% (2-tailed) to detect a moderate effect size (0.6 SDs) between groups. If the final sample size is 96 (assuming the majority of the additional patients are STEMI without DM group) the power will increase to between 85% and 90%.

Objective 1(c) will investigate the association between covariates and either number or migratory ability of CPCs in all participants. The primary analysis will be a mixed model regression with the Day 0 and Day 4 values modelled jointly. Calculation of the sample size needed to detect a statistically significant effect of a key predictor in such analyses requires a number of parameters to be estimated. These parameters are uncertain but plausible estimates have been assumed for illustration (scenarios 1 to 3), with 80% power:

	Scenario 1	Scenario 2	Scenario 3
r-squared for full model	0.22	0.24	0.20
r-squared for reduced model	0.14	0.16	0.11
number of predictors in full model	3	3	3
number of predictors being tested	1	1	1
**target sample size**	**78**	**77**	**72**
**power if sample size=96**	**87%**	**88%**	**90%**

Objective 2 will investigate the association between either the number or migratory ability of CPCs and myocardial scar size in all participants. The primary analysis will again be a mixed model regression. As illustrated in the above table, the sample size of 80 will allow a change in r-squared of 0.08 for a key predictor to be detected with 80% power. Power would increase from 80% to 87%-90% with a final sample size of 96.

7 Study procedures

7.1 Patient presentation

Patients presenting with suspected acute coronary syndromes (ACS) arrive in the Bristol Heart Institute by one of five mechanisms; referral from Accident & Emergency (A&E), referral from general practice, via the medical admissions unit, transfer from a peripheral hospital for specialist care, or directly to the catheterisation laboratory for primary PCI.

7.1.1 STEMI presentation

Troponin positive status can be assumed for patients confirmed as having had a STEMI. Demonstration of ST segment elevation on a 12-lead ECG, in the context of an acute presentation with chest pain, represents an acute STEMI, indicating occlusion of a coronary artery and consequent myocardial ischaemia. Patients presenting with these symptoms to the

BRI A&E or by a rapid response paramedic unit with a confirmed STEMI are transferred directly to the catheterisation laboratory for primary PCI.

7.1.2 NSTEMI presentation

Patients presenting with either a history of chest pain, or an ischaemic ECG (not ST elevation) and evidence of an elevated troponin 12 hours after symptom onset, are defined as suffering a NSTEMI. This is confirmed by a point of care troponin test on admission to cardiology. Troponin elevation may occur up to 12 hours after the onset of symptoms so serial tests may be needed.

Patients presenting via A&E or the medical admissions unit will be assessed by a cardiologist, on admission to hospital and transferred to dedicated cardiology beds in the Bristol Heart Institute (BHI), as soon as possible.

7.2 Standard treatment by the coronary care team

7.2.1 STEMI participants

Reopening of the occluded coronary artery can be achieved by pharmacological thrombolysis or mechanical disruption of the occlusion/clot utilising PCI. Primary PCI is superior to thrombolysis and has been adopted as the first line treatment for all patients presenting to the BRI with STEMI.

7.2.2 NSTEMI participants

Treatment involves anti-thrombotic therapy, initiation of cardioprotective medication (betablockers and ACE inhibitors), risk factor modification and early angiographic assessment (within 24-48hrs).

Patients referred from peripheral hospitals are usually assessed by a cardiology registrar on arrival and usually proceed to angiography within 24hrs.

7.2.3 Post operation inpatient care

After PCI or coronary angiography, patients remain in the coronary care unit or a cardiology ward for ongoing care. Time to discharge is usually 1 to 4 days, but this depends on how soon after admission the required procedures are carried out (delayed for NSTEMIs), the complexity & success of the procedure and the extent of damage sustained during the index event.

Inpatient treatment involves starting cardiac medication and increasing medication as required, assessment of left ventricular function with echocardiography, and introduction to the cardiac rehabilitation team.

The cardiac rehabilitation team will carry out a post operation clinical assessment and will monitor the patient response to treatment through clinical assessments until discharge. Routine clinical assessments include recording: heart rate, blood pressure and weight and undergo a 12-lead electrocardiogram.

7.2.4 Follow up patient care

Patients are given a follow up clinic appointment four to six weeks after discharge. At this appointment, patients will routinely have a set of observations recorded (heart rate, blood pressure and weight) and undergo a 12-lead electrocardiogram. A member of the cardiology team will take a history from the patient, focusing on any adverse events, hospital admissions, or changes to medication occurring since the index admission. Additionally, a cardiovascular examination will be performed.

7.3 Research procedures

7.3.1 Inpatient procedures

On Day 0 we will obtain a 10ml blood sample either at the time of PCI or coronary angiography or by venepuncture. On Day 0, after obtaining consenting, information required for the study will be collected on a baseline CRF; this will include participant contact details, demographic information, medical history and concomitant medication.

We will draw a second blood sample of 50mls on Day 4 (84 to 132 hours after the onset of symptoms) by venepuncture. Before discharge we will ask patients to undergo an MRI scan. This will be time-tabled up until 5 days after onset of symptoms. For those participants who are discharged without a Day 4 blood sample we will ask them to either return as an out-patient to the cardiac clinic at 84 to 132 hours after onset of symptoms or offer them a home visit by a Research Nurse who will collect and transfer the blood samples to CTEU for analysis.

7.3.2 Follow up visit at Day 28

Patients will attend for a standard out-patients clinic visit, where a clinical assessment focusing on patients' medications, adverse events and any hospital re-admission(s) will be performed. Patients who either fail to attend their scheduled appointment or attend their follow-up visit at another hospital, will be followed-up by telephone.

7.3.3 Follow up visit at 3 months

Participants will return at 3 months after the index admission for a research follow up visit. At this visit, they will undergo an MRI scan (lasting about 30 – 45 mins). A member of the research study team will take a history from the participants, focusing on any adverse events, hospital admissions, or changes to medication occurring since the index admission. Again, patients who fail to attend their scheduled appointment will be followed-up by telephone.

7.3.4 Follow up at 12 months

Participants will be contacted by a member of the research team by telephone at 12 months. The telephone interview will focus on any adverse events, hospital admissions or changes to medication occurring since the index admission.

7.4 Duration of study

The full study is planned to last from September 2009 until February 2014 (recruitment stopping in November 2012). This will include the set up, recruitment, follow up and write up of the study.

7.5 Data collection

The following data will be collected at the following time-points:

Data item	Admission	Day 0	Day 4	Day 28	3 Months	12 Months
Consent and Eligibility forms		✓				
Baseline CRF		✓				
Blood sample		✓	✓			
MRI Scan			✓+		✓	
Clinical assessment	✓	✓	✓	✓++	✓++	✓++

+data collected between Day 0 and Day 5

++data on medications, adverse events, re-admission(s) to hospital(s)

7.6 Source data

The following will be considered as source data:

- participant medical notes will be the source for participant contact details, medical history and routinely collected clinical data and the clinical assessments;
- NHS laboratory databases will be the source data for the troponin I, hsCRP, eGFR, $HbA1_c$, Hb and routine NHS laboratory test results;
- University laboratory databases will be the source for other study specific biochemical markers including number, migratory ability and viability of CPCs;
- The NHS laboratory database holding MRI scans will be considered the source data for all MRI images; the source for measurements derived from the images will be the relevant CRFs.

7.7 Screening and eligibility assessment

Researchers will consider the screening criteria described in Section 6.5 before approaching the patient with a Participant Information Sheet (PIS) describing the study.

7.8 Laboratory measurements of CPC characteristics

7.8.1 Migratory ability of CPCs

The migratory capacity of CPCs will be determined by a migration assay of CPCs with response to SDF-1, NGF or PBS (the SDF-1 and NGF vehicle) using Boyden chambers. Briefly, peripheral blood cells will be first submitted to separation steps to eliminate blood red cells. Then, a specified number of MNCs (which represent the monocyte fraction enriched for CPCs) will be placed in the upper part of a multiwell migration chamber. The lower part will contain basal medium supplemented with SDF-1, NGF or PBS. After an overnight incubation, cells remaining in the upper side and cells migrating to the lower side of the chamber will be collected and characterised by flow cytometry.

7.8.2 Number of CPCs

Blood samples will be stained with selected stem cell antibodies as described earlier. Counting beads will also be added in order to normalise the percentage of CPCs after FACS analysis. After staining with antibodies and washing, samples will be fixed with 2% PFA and stored in an allocated fridge within one week before FACS using a BD Canto II. Data will be analysed using FACSDiva software to estimate cell viability, percentage or absolute number of cells against selected CPC markers.

7.8.3 Viability of CPCs

CPCs will be studied by co-staining cells with the apoptosis marker Annexin-V-FITC and the necrosis marker 7-AAD.

7.9 Clinical assessments

These assessments will be carried out as routine care for STEMI and NSTEMI cohorts during the index admission and follow-up at 28 days (by clinic appointment or telephone). At 3 months after the index admission, participants will be invited to an out-patient clinical examination and the same assessment will be carried out. At 12 months after the index admission a member of the research team will contact the patient by telephone focusing on any adverse events, hospital admissions, or changes to medication since the index admission.

7.10 Magnetic Resonance Imaging (MRI) scans

7.10.1 MRI study procedures

Patients will undergo two MRI scans, the first at baseline (between Day 2 and Day 5) and the second 3 months after the index admission. All MRI scans will be performed on a 1.5T scanner

(Magnetom Avanto; Siemens; Elangen, Germany), with surface phased array received coil and retrospective electrographic triggering. Myocardial perfusion and previous infarction will be assessed using adenosine and gadolinium-DTPA first-pass data acquisition and late-enhancement imaging.

After obtaining scout images, cine steady-state free precession (SSFP) CMR images will be acquired during short breath holds in multiple long axis (2-, 3- and 4-chamber planes) and multiple short-axis images, encompassing the left ventricle from the base to the apex. Depending on the length of the ventricle a variable number of short axis slices will be obtained (generally ranging from 7 to 12 slices).
Typical cine SSFP images parameters will be: repetition time (TR) 39.3 ms, echo time (TE) 1.1 ms, flip angle 75°, 8-mm slice thickness, no interslice gap, matrix of 256 x 256, field of view ranging from 340 to 400 mm, and a voxel size of approximately 1.7 x.7 x 8.0 mm.
For T2w-STIR imaging, a breath-hold black-blood segmented turbo spin echo technique with a triple inversion recovery preparation module (TR 700ms double gated, TE 59 ms, flip angle 180°, TI 170 ms, slice thickness 8 mm, no interslice gap, field of view 340 to 400 mm, matrix 256 x 256, and a voxel size of 2.3 x 1.3 x 8 mm) will be adopted. Each slice was obtained during an end-expiratory breath-hold of 12 to 15 s, depending on the patient's heart rate.
Rest myocardial perfusion will be evaluated with a first pass technique using a single shot spoiled gradient echo pulse sequence (slice thickness 10 mm) during 60 consecutive heartbeats. Three short-axis slices (basal, mid cavity, and apical levels) will be acquired injecting 0.1 mmol/kg of gadolinium-chelate contrast agentat 3 ml/s followed by 20 ml saline flush in the right anterocubital vein.
Finally, long- and short-axis late enhancement images (LGE) will be obtained by using a segmented inversion recovery technique and acquired 10 to 15 min after contrast injection. Sequence parameters will be the following: TR 700 ms, TE 3.19 ms, matrix 256 x 256, flip angle 25°, slice thickness 8.0 mm, no interslice gap, and voxel size 1.7 x1.4 x 8 mm. The inversion time will be progressively optimized to null the signal in the normal myocardium (typical values, 250 to 350 ms).
To ensure matching slice position, cine, T2w-STIR, and LGE images will be acquired at exactly the same slice position.

7.10.2 MRI outcome definitions

Analysis of the imaging protocol will allow accurate and reproducible calculation of the infarct size, end-diastolic and and-systolic volumes, left ventricular (LV) mass, stroke volume, ejection fraction, aortic pulsatility and pulse wave velocity as well as myocardial perfusion. These are described in more detail as follows:

- Regional LV thickening of the 'affected' segments 3 months after surgery, i.e. end systolic thickness minus end diastolic thickness (millimetres). Affected segments will be those scored on the baseline cardiac MRI as 1-5 () on a 5 point scale. Changes in regional LV wall function will be measured in different ways. LV wall thickening will be determined in all segments; LV wall thickness measurements will be recorded from end-systolic and end-diastolic images. Secondary measures of wall thickening will include standard wall score index (WMSI); wall motion for each segment will be rated from cine MRI images on a 5-point scale (0, normal; 1, mild hypokinesia; 2, severe hypokinesia; 3, akinesia; 4, dyskinesia).
- LV regional myocardial viability will be measured using gadolinium contrast images. Infarct size will be measured as a planimetered area (and volume derived by multiplying area by slice thickness) and volume fraction of enhanced myocardium. For myocardial viability analysis, delayed-enhancement images for each segment will be scored as for function analysis, and quantified by computer-assisted planimetry on short-axis images. Segments will be graded in transmural extent on a 5-point scale (0, no HE; 1, hyper-enhancement extending from 1% to 25%; 2, 26% to 50%; 3, 51% to 75%; 4, hyperenhancement >75% of LV wall thickness for that segment).

- End systolic volume, stroke volume and ejection fraction will be measured according to the validated MRI laboratory standards, using contiguous short axis slices obtained by cine MRI with correction for long axis motion (Argus 4D Software or Brompton). End-diastolic and end-systolic endocardial traces will be used to determine end-diastolic and end-systolic LV volumes and total ejection fraction.

7.11 Planned recruitment rate

The study plans to recruit between 80 and 96 participants (see section 6.6). The different size of the four cohorts reflects the frequency of presentation of different kinds of patient and the clinical nature of NSTEMI. We expect to recruit into the non-DM STEMI cohort relatively quickly, but more slowly into the other three cohorts. Consequently, recruitment may take up to 3 years. Overall, we expect to recruit about 10 to 20% of eligible patients presenting during the time windows when recruitment is feasible.

7.12 Discontinuation/withdrawal of participants from study treatment

We anticipate a follow up rate at least 90% for the 3 month MRI scans. Appointments for these visits will be booked at the Day 28 visit. Participants who fail to attend the Day 28 visit will be contacted by telephone in order to collect the follow-up data and also to arrange the 3 month follow up visit; this will happen rarely because Day 28 visits are routinely re-booked if patients fail to attend. All patients will be reminded of their 3 month appointment by letter.

Participants who fail to attend the 3 month follow up visit will be contacted by telephone in the week following the missed appointment to arrange an alternative appointment within the following two weeks. These patients will then receive a follow up letter to remind them about their appointment.

7.13 Frequency and duration of follow up

The duration of follow-up after the index event/presentation will be 12 months. During this period, patients will be followed up on 4 occasions:

- Day 4 (in hospital for most patients, but some participants may be discharged early and need to return or they will be offered a home visit by a Research Nurse)
- Day 28 (standard out-patient clinic visit only or telephone follow-up)
- 3 months (research out-patient visit for all patients or telephone follow-up)
- 12 months (telephone follow-up)

7.14 Expenses and benefits

A limited number of participants taking part in this study will be discharged before Day 4. Participants will be asked to either return between 84 to 132 hours after onset of symptoms for the second blood sample or they will be offered a home visit by a Research Nurse who will collect and transfer the blood samples to CTEU for analysis. It is anticipated that all participants will return for a 3 month follow up visit.

Reasonable travel expenses incurred by participants for these visits will be reimbursed.

7.15 Measures taken to avoid bias

Ideally, this study would recruit consecutive eligible patients to maximise the representativeness of the study sample. This is not feasible for logistical reasons. Nevertheless, since we expect to recruit consecutive eligible patients who consent during the time window for recruitment allowed by available research staffing, we do not believe that the representativeness of the study sample will be compromised. In the event of staff absences or equipment down-time, recruitment will be suspended preventing selective recruitment.

Laboratory measurements of CPC characteristics will be made without knowledge of the STEMI/NSTEMI and DM status of the participant from whom the blood sample was obtained (Section 7.8). Similarly, the outcome assessor scoring of MRI outcomes will be blinded.

Attrition bias will be minimised by the methods described under Section 7.12.

7.16 Analyses

This section will be clarified and expanded in a dedicated statistics analysis plan (SAP), written before carrying out any comparative / inferential analyses and based on the standard operating procedure of the CTEU for writing and approving a SAP. The SAP will be written by a statistician during the data collection period and it will be approved by a senior statistician before database lock and will include all analyses to be carried out.

7.16.1 Statistical analyses of primary CPC outcomes

Objectives 1(a), 1(b) and 1(c) will be analysed by the following methods.

Descriptive statistics will be used to summarise the data both overall and for separate cohorts. Estimates and 95% confidence intervals on log scales will be back-transformed to estimate the geometric means and 95% confidence intervals on the raw measurement scales.

A general linear model will be used to compare CPC responses. The CPC outcome measure will be assessed and an appropriate transformation will be applied to normalise the distribution, if necessary. The data are likely to be positively skewed. On this assumption, the data will be log-transformed. For each objective, a model will be fitted including the following covariates: the patient age; gender and, for each blood sample, the number of hours since onset of symptoms.

For objective 1(a) and 1(b), we will estimate the effect of STEMI vs. NSTEMI status, the effect of DM vs. non-DM status, with 95% confidence intervals in a single model, with the covariates described above. The interaction term for these two factors will be included in the model and tested, but will only be retained in the model if it is statistically significant at the 5% level. For objective 1(c), the peak troponin value available (again, transformed if appropriate) will be fitted instead of STEMI vs. NSTEMI status; the regression coefficent for the CPC outcome and its confidence interval for a unit change in troponin will be estimated, adjusted for the covariates described above. A similar model will be fitted substituting HbA1c for DM vs. non-DM status. Finally, a model fitting both troponin, HbA1c, the interaction of these two variables and covariates will be fitted; the interaction will only be retained in the model if it is statistically significant at the 5% level.

Similar methods will be used for analyses of secondary outcomes.

7.16.2 Statistical analyses of primary MRI outcomes

We propose to carry out a two-stage analysis of segmental data to compare scores between the STEMI/NSTEMI groups: (a) segments shown to have poor function at baseline (‘affected’ segments); (b) all segments. At each stage, scores for segments included in the analysis will be expressed as a percentage of the maximum available score if all included segments were functioning optimally. Although each segment rating is ordinal, percentage scores will be treated as continuous when comparing between STEMI groups. For all outcomes, mean scores (±SD) for each arm (transformed if necessary) will be described.

We plan to use general linear models to fit the outcomes from the MRI scan. These models will be analogous to those fitted to address objective 1(c). The outcome will be the MRI outcome of interest, i.e. myocardial scar size, and the covariates of primary interest will be the CPC outcomes (number, migratory ability and viability).

7.16.3 Analysis of the clinical outcomes

We will use descriptive statistics to summarise clinical outcomes. No inferential statistical analysis of clinical outcomes is planned.

7.17 Subgroup analyses

We aim to estimate the effects of STEMI/NSTEMI and DM status in a single linear model. No other subgroup analyses based on the characteristics of participants are planned.

7.18 Frequency of analyses

There will be no interim analyses. However, in order to write the analysis plan, the study statistician will have access to a datafile containing the CPC and MRI outcome but without information about STEMI/NSTEMI and DM status and covariates. The statistician will also have access to a datafile containing key covariates but without the outcome data.

7.19 Criteria for the termination of the study

Each participant has the right to withdraw at any time. If a patient wishes to withdraw post-operatively we will continue to analyse any data already collected, unless the patient expresses a wish for their samples and any associated data to be destroyed.

8 Project management

The study will be managed by the Clinical Trials and Evaluation Unit (CTEU) at the Bristol Heart Institute (epidemiologist/trialist, Prof B Reeves; statistician, Dr C Rogers). The CTEU will prepare all the study documentation and data collection forms, monitor recruitment, enter data and check data quality as the study progresses and carry out study analyses in collaboration with the clinical investigators.

9 Steering committee

This study will be overseen by the Cardiovascular Research Board (CRB) of the Hospitals Bristol NHS Foundation Trust (UHBristol) and the University of Bristol (the “Partnership”). The CRB is made up of representatives from the Partnership and has been established to oversee cardiovascular projects being carried out by the Partnership (primarily, component projects of National Institute for Health Research (NIHR) Applied Programme Grants, the NIHR Cardiovascular Biomedical Research Unit (BRU)). The Board will act as the Steering Committee for this and other single centre studies in these NIHR programmes. The Board is chaired by the Director of the BRU, Professor Angelini. Study coordinators assigned to work on the study will work closely with the Principal Investigator, who will report to the CRB.

There will be no Data Monitoring and Safety Committee for this cohort study. Research procedures required for the research (venepuncture to obtain blood samples and MRI scans) are standard health care practices. Adverse events arising from these investigations will be reported periodically to the CRB.

10 Safety reporting

Serious and other adverse events will be recorded and reported in accordance with the International Conference for Harmonisation of Good Clinical Practice (ICH GCP) guidelines and the Sponsor’s Research Related Adverse Event Reporting Policy. In cardiology, transient complications are not unexpected and are not infrequent. The research team will only notify Sponsor for Serious Adverse Events (SAEs) deemed to be related to venepuncture and MRI scanning and/or fatal and life-threatening SAEs. The Sponsor will inform CTEU Bristol if the event needs to be reported to the REC (usually only events that are both related to the intervention and unexpected).

10.1 Period for recording adverse events

Data on adverse events will be collected from consent and for the 12-month follow-up period.

11 Ethical considerations

11.1 Ethical review

Ethics review of the protocol for the study and other study-related essential documents (e.g. PIS, consent form) will be carried out by a UK NHS Research Ethics Committee (REC) and other bodies with similar roles/authority for centres outside the UK. This study does not raise any substantive ethical issues since participants will be receiving standard NHS care and having research investigations that carry very little risk.

11.2 Obtaining informed consent from participants

Our primary concern will be not to approach a patient until his or her condition has stabilised and the attending cardiologist considers that it is appropriate to describe the study.

11.2.1 STEMI participants

Participants will undergo PCI immediately after admission to cardiology. Then a member of the clinical care member of staff will inform the participant about the study and give the PIS to the participant. The participant will be given time to read the PIS and discuss their participation with others outside the research team (e.g. relatives or friends) if they wish. Most participants will have at least 2 hours from the time they receive the PIS to consider whether to participate or not, however, for participants presenting late after onset of symptoms, the time available may be less. A clinical member of staff will address any questions the participant has about the study, ascertain whether he/she has had sufficient time to consider it and if so, invite him/her to give written informed consent. No patient will be recruited unless they feel they have had enough time to fully understand the study and are able to give informed consent.

11.2.2 NSTEMI participants

NSTEMI participants will be given the Participant Information Sheet (PIS) after admission and stabilisation. Participants will be approached for consent only when troponin positive ACS status has been met. The participant will be given time to read the PIS and discuss their participation with others outside the research team (e.g. relatives or friends) if they wish. Most participants will have at least 2 hours from the time they receive the PIS to consider whether to participate or not, however, for participants presenting late after onset of symptoms, the time may be less. They will then be invited to ask further questions about the study, confirm they have had sufficient time to consider the study and to give written informed consent. No patient will be recruited unless they feel they have had enough time to fully understand the study and are able to give informed consent.

12 Research governance

The University Hospitals Bristol NHS Foundation Trust (UHBristol) is the sponsor for the study.

This study will be conducted in accordance with:

- The European Union Directive 2001/20/EC on clinical trials;
- The Medicine for Human Use (Clinical Trial) Regulations 2004;
- International Conference for Harmonisation of Good Clinical Practice (ICH GCP) guidelines;
- Research Governance Framework for Health and Social Care.

12.1 NHS approval

The investigators will seek approval to carry out the study from the University Hospitals of Bristol NHS Trust. Local Research and Development (R&D) approval in the UK requires that the study be conducted in compliance with the Research Governance Framework.

12.2 Investigators' responsibilities

Investigators will be required to ensure that Local Ethics Committee and research governance approvals have been obtained and that any contractual agreements required have been signed off by all parties prior to the start of the study. Investigators will be required to ensure compliance to the protocol and study manual and with completion of the CRFs. Investigators will be required to allow access to study documentation or source data on request for monitoring visits and audits performed by the Sponsor or CTEU or any regulatory authorities.

12.3 Monitoring by sponsor

The study will be monitored and audited in accordance with the Sponsor's policy, which is consistent with the Research Governance Framework and the Medicines for Human Use (Clinical Trials) Regulations 2004. All study related documents will be made available on request for monitoring and audit by the sponsor (or CTEU if they have been delegated to monitor) or the Ethics Committee.

12.4 Indemnity

This is an NHS-sponsored research study. For NHS sponsored research, HSG(96)48 reference no.2 refers. If there is negligent harm during the clinical study when the NHS body owes a duty of care to the person harmed, NHS Indemnity covers NHS staff, medical academic staff with honorary contracts, and those conducting the study. NHS Indemnity does not offer no-fault compensation and is unable to agree in advance to pay compensation for non-negligent harm. Ex gratia payments may be considered in the case of a claim

13 Data protection and patient confidentiality

Data will be collected and retained in accordance with the UK Data Protection Act 1998.

13.1 Data handling

Data will be entered onto a database and data validation and cleaning will be carried out throughout the study.

13.2 Data storage

We will propose to the UK NHS REC that we retain all study documentation in a secure location during the conduct of the study and for 5 years after the end of the study, when all patient identifiable paper records will be destroyed by confidential means. In compliance with the MRC Policy on Data Preservation, we will also propose that the fully anonymised dataset, a separate secure electronic 'key' with a unique patient identifier, and relevant 'meta'-data about the study be retained in electronic form indefinitely because of the potential for the raw data to be used subsequently for secondary research.

13.3 Data sharing

Data will not be made available for sharing until after publication of the main results of the study. Thereafter, anonymised individual patient data will be made available for secondary research, conditional on assurance from the secondary researcher that the proposed use of the data is compliant with the MRC Policy on Data Preservation and Sharing regarding scientific quality, ethical requirements and value for money. We propose that a minimum requirement with respect to scientific quality should be a publicly available pre-specified protocol describing the purpose, methods and analysis of the secondary research, e.g. a protocol for a Cochrane systematic review. The second file containing patient identifiers would be made available for record linkage or a similar purpose, subject to confirmation that the secondary research protocol has been approved by a UK REC or other similar, approved ethics review body.

14 Dissemination of findings

The findings will be disseminated by usual academic channels, i.e. presentation at international meetings, as well as by peer-reviewed publications and through patient organisations and newsletters to patients, where available.

15 References

1. Asahara T, Murohara T, Sullivan A, et al. Isolation of putative progenitor endothelial cells for angiogenesis. *Science*. 1997;275:964-967.
2. Kalka C, Masuda H, Takahashi T, et al. Transplantation of ex vivo expanded endothelial progenitor cells for therapeutic neovascularization. *Proc Natl Acad Sci U S A.* 2000;97:3422-3427.
3. Walter DH, Haendeler J, Reinhold J, et al. Impaired CXCR4 signaling contributes to the reduced neovascularization capacity of endothelial progenitor cells from patients with coronary artery disease. *Circ Res*. 2005;97:1142-1151.
4. Krenning G, van der Strate B, Schipper M, et al. CD34(+) Cells Augment Endothelial Cell Differentiation of CD14(+) Endothelial Progenitor Cells in vitro. *J Cell Mol Med*. 2008.
5. He T, Peterson TE, Katusic ZS. Paracrine mitogenic effect of human endothelial progenitor cells: role of interleukin-8. Am J *Physiol Heart Circ Physiol*. 2005;289:H968-972.
6. Tepper OM, Galiano RD, Capla JM, et al. Human endothelial progenitor cells from type II diabetics exhibit impaired proliferation, adhesion, and incorporation into vascular structures. *Circulation*. 2002;106:2781-2786.
7. Caporali A, Pani E, Horrevoets AJ, et al. Neurotrophin p75 receptor (p75NTR) promotes endothelial cell apoptosis and inhibits angiogenesis: implications for diabetes-induced impaired neovascularization in ischemic limb muscles. *Circ Res*. 2008;103:e15-26.
8 Scachinger V., Birgit A.,... *et* al. Transplantation of progenitor cells and regeneration enhancement in acute myocardial infarction. *J Am Col Cardio Found.*, 2004; **44**.
9 Assmus B., Honold J.,… et al. Transcoronary transplantation of progenitor cells after myocardial infarction. *N Eng J Med*, 2006; 355:1222-32.
10 Elm E., Altman D., Egger M., Pocock S., Gøtzsche P. and Vandenbroucke P. The Strengthening the Reporting of Observational Studies in Epidemiology (STROBE) statement: guidelines for reporting observational studies. *The Lancet*, 2007; 370:1453-1457.

16 Amendments to the protocol

Amendment number	Previous version	Previous date	New version	New date	Brief summary of change	Date of ethical approval
8	8.0	21-Nov-2012	9.0	30-Aug-2013	• The option of a home visit for the collection of the Day 4 blood samples has been added	Date to be added once REC approval is obtained
7	7.0	10-May-2012	8.0	21-Nov-2012	• The 4 hour 'thinking time' has been amended<br>• Names of members of the Cardiovascular Research Board have been removed	Date to be added once REC approval is obtained
6	6.0	13-Apr-2011	7.0	10-May-2012	• Eligibility criterion rephrased<br>• Sample size calculation, research procedures, MRI scan and safety reporting sections updated	29-May-2012
5	5.0	15-Jan-2010	6.0	13-Apr-2011	• Change in the cohort number from 72 to 80 participants<br>• Definition of Day 4 window extended	11-Jul-2011
4	4.0	14-Dec-2009	5.0	15-Jan-2010	• Beta-2 is included an additional secondary marker<br>• MRI scans section updated	11-Feb-2010
2	3.0	03-Dec-2009	4.0	14-Dec-2009	• CD164 is included as an additional CPC marker	14-Jan-2010
1	2.0	21-Oct-2009	3.0	03-Dec-2009	• Clarification of secondary laboratory CPC markers<br>• Change in the amount of blood collected for analysis (from 5mls to 10mls and from 40mls to 50mls, on Days 0 and 4 respectively)	11-Dec-2009

Supplementary tables and figures

Supplementary Figure 1: Schema of the study

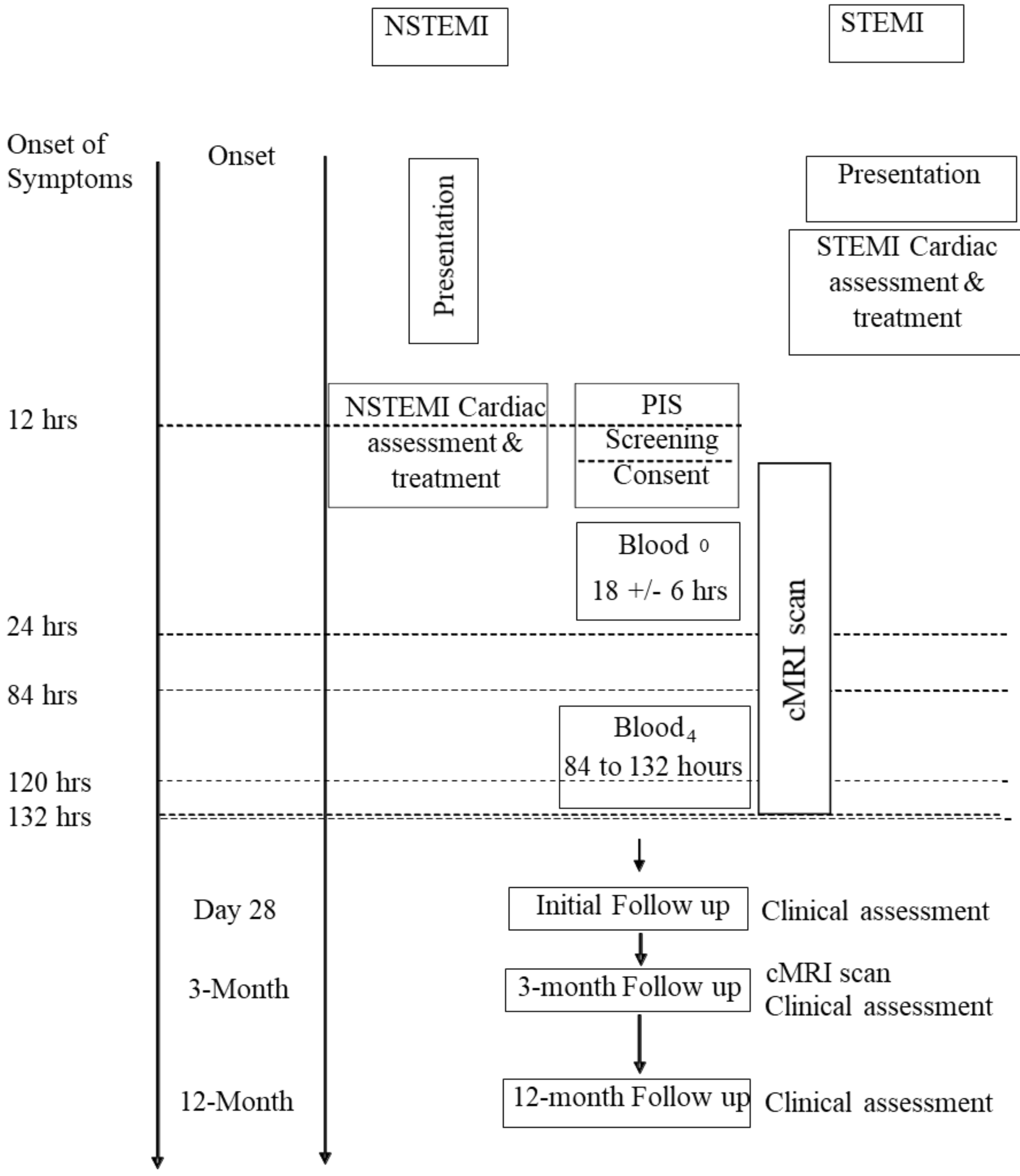

Supplementary Figure 2: Post-hoc analysis - associations between STEMI and diabetes with respect to percentage of CD34+/CXCR4+ on day 4 adjusting for CD34+/CXCR4+ on day 0

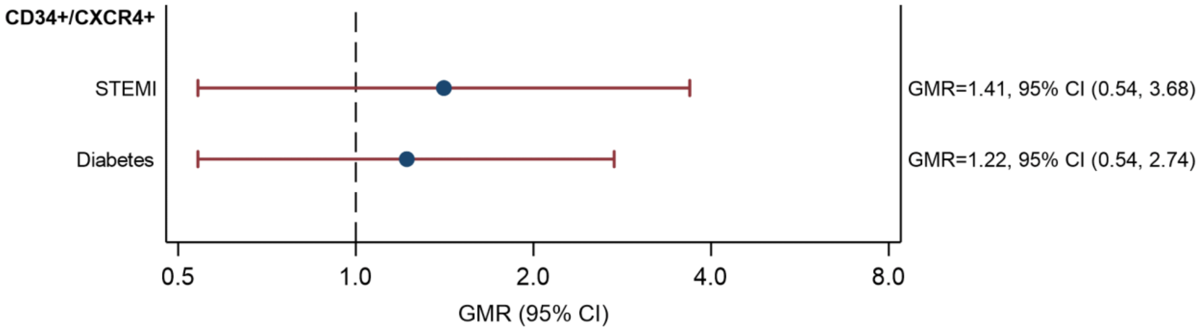

Notes:
Model fitted to participants with lab data available on day 0 and day 4: 55 participants (14 STEMI with DM, 29 STEMI without DM, 6 NSTEMI with DM, 6 NSTEMI without DM).
Estimates are adjusted for CD34+/CXCR4+ on day 0, age, sex and time since onset of symptoms.
*p-value for interaction STEMI*Diabetes: p=0.4650*
Abbreviations: *STEMI=ST-elevation myocardial infarction, NSTEMI=Non-ST-elevation myocardial infarction*

Supplementary Figure 3: Associations between Troponin T and CRP and the percentage of CPCs

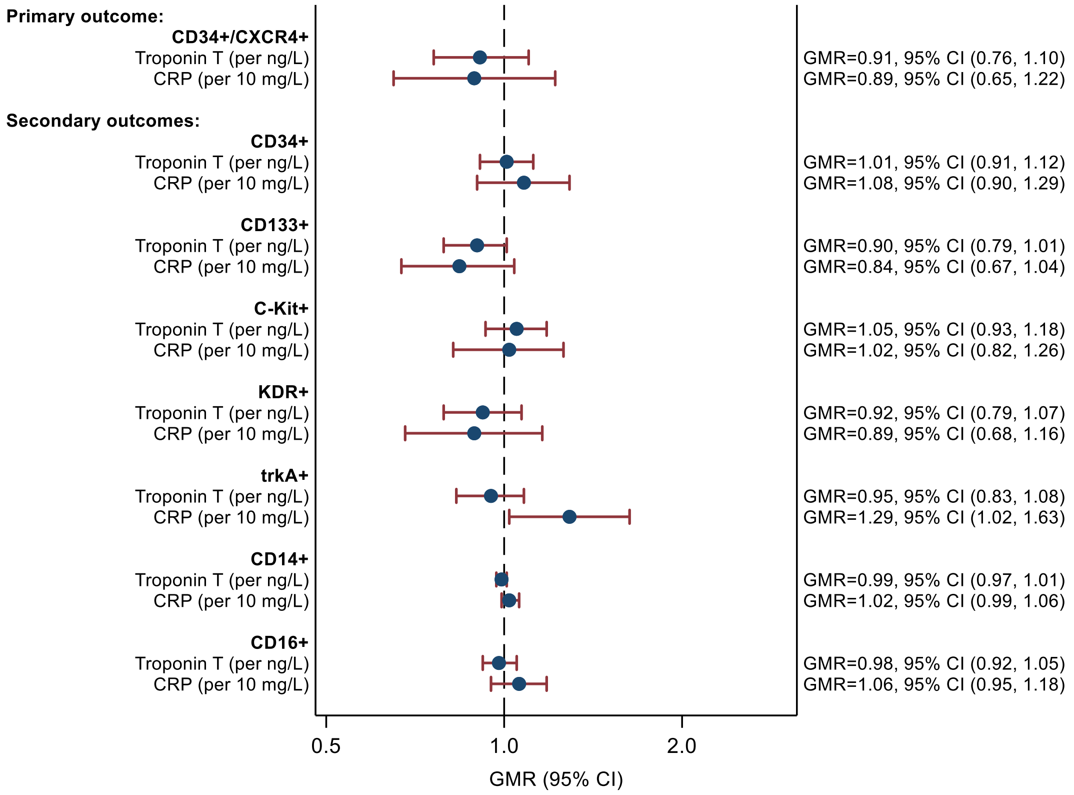

Notes:

Model fitted to participants with CPC data available and non-missing troponin T and CRP: 32 participants (10 STEMI with DM, 16 STEMI without DM, 4 NSTEMI with DM, 2 NSTEMI without DM)

Estimates are adjusted for diabetes, age, sex and time since onset of symptoms.

*p-values for interaction troponin T*time: CD34+/CXCR4+ (p=0.7715), CD34+ (p=0.4285), CD133+ (p=0.6827), C-Kit (p=0.4283), KDR+ (p=0.1246), trkA+ (p=0.2542), CD14+ (p=0.4395), CD16+ (p=0.9053)*

*p-values for interaction CRP*time: CD34+/CXCR4+ (p=0.3550), CD34+ (p=0.4028), CD133+ (p=0.6112), C-Kit (p=0.8424), KDR+ (p=0.9955), trkA+ (p=0.8769), CD14+ (p=0.3905), CD16+ (p=0.4676)*

Abbreviations: *STEMI=ST-elevation myocardial infarction, NSTEMI=Non-ST-elevation myocardial infarction, CRP=C-reactive protein, CI=Confidence interval, GMR=Geometric mean ratio*

Supplementary Table 1: Time points of data collection

Data item	Admission	Day 0	Day 4	Day 28	3 Months	12 Months
Consent and Eligibility forms		✓				
Baseline CRF		✓				
Blood sample		✓	✓			
MRI Scan			✓+		✓	
Clinical assessment	✓	✓	✓	✓++	✓++	✓++

+data collected between Day 0 and Day 5

++data on medications, adverse events, re-admission(s) to hospital(s)

Supplementary Table 2: Details of withdrawals

Withdrawal ID	Cohort	Who withdrew the patient	Reason for withdrawal (free text)	Days from recruitment to withdrawal	Willing for follow-up to continue	Willing for passive data collection to continue	Willing for data collected already to be used
1	STEMI, without DM	Clinician	Post procedural complications secondary to a significant GI bleed requiring OGD intervention and subsequent blood transfusion.	3	Yes	Yes	Yes
2	NSTEMI, without DM	Clinician	Patient required surgery so therefore missed MRI scan. Day 4 bloods were taken but due to power cut/floods samples could not be processed; fridge failed.	3	Yes	Yes	Yes

Abbreviations: *DM=Diabetes Mellitus, STEMI=ST-elevation myocardial infarction, NSTEMI=Non-ST-elevation myocardial infarction, GI= Gastrointestinal, OGD= Oesophago-Gastro- Duodenoscopy, MRI= Magnetic resonance imaging*

Supplementary Table 3: Protocol deviations

Protocol deviation	STEMI with DM (n = 16)	STEMI without DM (n = 34)	NSTEMI with DM (n = 8)	NSTEMI without DM (n = 13)	Overall (n = 71)
Patient did not proceed to angiography within the allowed time window after the onset of symptoms*	0/16 (0.0%)	0/34 (0.0%)	3/8 (37.5%)	5/13 (38.5%)	8/71 (11.3%)
Day 0 blood sample collected >24h after patient consented	0/16 (0.0%	0/34 (0.0%)	0/8 (0.0%)	0/13 (0.0%)	0/71 (0.0%)
Day 4 blood sample not collected within the allowed time window after onset of symptoms**	5/16 (31.3%)	5/32 (15.6%)	2/8 (25.0%)	3/13 (23.1%)	15/69 (21.7%)
cMRI scan at baseline not done within 5 days since onset of symptoms***	1/14 (7.1%)	1/29 (3.4%)	1/6 (16.7%)	2/11 (18.2%)	5/60 (8.3%)
cMRI scan at follow-up was not done within 3 months since index admission****	0/11 (0.0%)	3/27 (11.1%)	0/8 (0.0%)	1/10 (10.0%)	4/55 (7.3%)

Notes:
** Allowed time window: within 48 hours. Hours between onset of symptoms and angiography if >48 hours: 68 (n=1), 70 (n=1), 78 (n=1), 80 (n=1), 84 (n=1), 93 (n=1), 128 (n=1), 221 (n=1)*
*** Allowed time window: 84 hours to 132 hours. Hours between onset of symptoms and day 4 sample if outside allowed time window: 68 (n=1), 72 (n=1), 75 (n=2), 77 (n=1), 78 (n=1), 80 (n=1), 82 (n=1), 83 (n=2), 136 (n=1), 140 (n=2), 142 (n=1), 153 (n=1)*
**** Days between onset of symptoms and baseline cMRI if >5 days: 6 (n=3), 7 (n=1), 8 (n=1)*
***** 14-day time window allowed. Months between index admission and cMRI follow-up scan if >3.5 months: 3.55 (n=1), 3.62 (n=1), 4.18 (n=1), 4.67 (n=1)*
Abbreviations: *DM=Diabetes Mellitus, STEMI=ST-elevation myocardial infarction, NSTEMI=Non-ST-elevation myocardial infarction*

Supplementary Table 4: Perioperative lab measurements on day 0*

	STEMI with DM (n = 16)	STEMI without DM (n = 34)	NSTEMI with DM (n = 8)	NSTEMI without DM (n = 13)	Overall (n = 71)
**Perioperative samples**					
Total Cholesterol (mmol/L) [a]	5.0 (3.2, 6.7)	5.3 (4.8, 6.3)	3.6 (3.4, 3.8)	5.3 (5.0, 6.1)	5.2 (4.3, 6.1)
CRP (mg/L) [b]	8.5 (4.0, 10.5)	10.0 (5.0, 10.0)	10.0 (3.0, 10.0)	8.5 (3.0, 13.0)	9.0 (4.0, 10.0)
Creatinine (µmol/L) [c]	82.1 (20.4)	93.3 (17.0)	92.3 (25.8)	86.5 (18.3)	89.5 (19.2)
eGFR (ml/min/1.73^2) [d]	89.5 (83.5, 90.0)	81.0 (75.0, 90.0)	83.0 (65.0, 90.0)	79.0 (71.0, 89.0)	83.0 (75.0, 90.0)
K+ (mmol/L) [e]	4.3 (0.5)	4.3 (0.4)	3.9 (0.6)	4.1 (0.4)	4.2 (0.4)
Na+ (mmol/L) [f]	137.5 (3.3)	138.6 (3.2)	138.0 (2.3)	138.8 (1.5)	138.3 (2.9)
Urea (mmol/L) [g]	6.3 (2.1)	5.6 (1.8)	6.4 (2.6)	5.6 (1.7)	5.9 (2.0)
PLT (x10^9/L) [h]	223.6 (56.7)	234.9 (50.0)	231.5 (42.7)	221.1 (92.7)	229.7 (58.2)
RBC (x10^9/L) [i]	4.8 (0.6)	4.7 (0.4)	4.2 (0.6)	4.8 (0.3)	4.7 (0.5)
WBC (x10^9/L) [j]	11.3 (3.8)	11.0 (3.3)	8.4 (1.7)	9.8 (3.2)	10.6 (3.3)
Glucose (mmol/L) [k]	15.6 (11.4, 23.0)	6.5 (5.5, 7.6)	8.8 (7.9, 12.5)	5.8 (5.5, 6.1)	6.9 (5.7, 11.3)
ALT (IU/L) [l]	37.0 (30.0, 45.5)	36.0 (24.0, 60.0)	34.0 (30.0, 47.0)	24.0 (19.0, 35.0)	34.0 (25.0, 46.0)
MgSo4- (mmol/L) [m]	0.8 (0.1)	0.8 (0.1)	0.8 (0.0)	-	0.8 (0.1)
Troponin I (ng/L) [n]	24.5 (0.0, 49.0)	56.5 (26.0, 77.0)	-	-	53.0 (25.0, 75.5)
Troponin T (ng/L) [o]	2323.0 (1592.5, 3120.5)	1166.5 (676.5, 3188.5)	332.0 (101.5, 949.0)	167.0 (66.0, 275.0)	890.0 (216.5, 2241.0)

Notes:

** If more than one sample taken on day 0, the maximum value of each measure is taken. If no sample was taken on date of consent then sample on day nearest 0 was used.*

Data are presented as median (IQR) or mean (SD)

Missing data (STEMI with DM, STEMI without DM, NSTEMI with DM, NSTEMI without DM):

[a] Data missing for 28 patients (5, 15, 4, 4)

[b] Data missing for 12 patients (0, 4, 3, 5)

[c] Data missing for 4 patients (0, 0, 1, 3)

[d] Data missing for 5 patients (0, 1, 1, 3)

[e] Data missing for 5 patients (0, 1, 1, 3)

[f] Data missing for 5 patients (0, 1, 1, 3)

[g] Data missing for 4 patients (0, 0, 1, 3)

[h] Data missing for 6 patients (0, 1, 2, 3)

[i] Data missing for 6 patients (0, 1, 2, 3)
[j] Data missing for 6 patients (0, 1, 2, 3)
[k] Data missing for 26 patients (4, 11, 5, 6)
[l] Data missing for 22 patients (4, 9, 3, 6)
[m] Data missing for 38 patients (6, 13, 6, 13)
[n] Data missing for 59 patients (14, 24, 8, 13)
[o] Data missing for 19 patients (4, 14, 0, 1)
Abbreviations: *DM=Diabetes Mellitus, STEMI=ST-elevation myocardial infarction, NSTEMI=Non-ST-elevation myocardial infarction, CRP=C-reactive protein, eGFR=Estimated Glomerular Filtration Rate, PLT=Platelet count, RBC=Red blood cells, WBC=White blood cells, ALT=Alanine transaminase*

Supplementary Table 5: Medications at hospital discharge and during follow-up

Medication	STEMI with DM (n = 16)	STEMI without DM (n = 34)	NSTEMI with DM (n = 8)	NSTEMI without DM (n = 13)	Overall (n = 71)
**Medications at discharge**					
Aspirin	16/16 (100.0%)	33/33 (100.0%)	8/8 (100.0%)	13/13 (100.0%)	70/70 (100.0%)
Clopidogrel	5/16 (31.3%)	14/33 (42.4%)	6/8 (75.0%)	10/13 (76.9%)	35/70 (50.0%)
Prasugrel	11/12 (91.7%)	19/19 (100.0%)	0/2 (0.0%)	2/5 (40.0%)	32/38 (84.2%)
Warfarin	1/16 (6.3%)	1/33 (3.0%)	1/8 (12.5%)	1/13 (7.7%)	4/70 (5.7%)
Heparin	1/16 (6.3%)	0/33 (0.0%)	0/8 (0.0%)	0/13 (0.0%)	1/70 (1.4%)
Clexane	0/16 (0.0%)	0/33 (0.0%)	1/8 (12.5%)	0/13 (0.0%)	1/70 (1.4%)
Beta-blockers	14/16 (87.5%)	31/33 (93.9%)	8/8 (100.0%)	12/13 (92.3%)	65/70 (92.9%)
Calcium channel antagonists	2/16 (12.5%)	3/33 (9.1%)	0/8 (0.0%)	0/13 (0.0%)	5/70 (7.1%)
Nitrates	1/16 (6.3%)	2/33 (6.1%)	0/8 (0.0%)	1/13 (7.7%)	4/70 (5.7%)
Potassium channel activators	0/16 (0.0%)	0/33 (0.0%)	0/8 (0.0%)	1/13 (7.7%)	1/70 (1.4%)
Lipid lowering agent	16/16 (100.0%)	33/33 (100.0%)	8/8 (100.0%)	13/13 (100.0%)	70/70 (100.0%)
ACE inhibitors	12/16 (75.0%)	30/33 (90.9%)	7/8 (87.5%)	13/13 (100.0%)	62/70 (88.6%)
Angiotensin-|| antagonists	4/16 (25.0%)	0/33 (0.0%)	1/8 (12.5%)	0/13 (0.0%)	5/70 (7.1%)
Loop diuretics	0/16 (0.0%)	1/33 (3.0%)	3/8 (37.5%)	0/13 (0.0%)	4/70 (5.7%)
Thiazide diuretics	2/16 (12.5%)	0/33 (0.0%)	0/8 (0.0%)	0/13 (0.0%)	2/70 (2.9%)
Digoxin	0/16 (0.0%)	0/33 (0.0%)	0/8 (0.0%)	0/13 (0.0%)	0/70 (0.0%)
Amiodrone	0/16 (0.0%)	0/33 (0.0%)	0/8 (0.0%)	0/13 (0.0%)	0/70 (0.0%)
Thyroxine	1/16 (6.3%)	0/33 (0.0%)	0/8 (0.0%)	0/13 (0.0%)	1/70 (1.4%)
Metformin	8/16 (50.0%)	0/33 (0.0%)	2/8 (25.0%)	0/13 (0.0%)	10/70 (14.3%)
Sulphonylureas	1/16 (6.3%)	1/33 (3.0%)	1/8 (12.5%)	0/13 (0.0%)	3/70 (4.3%)
Thiazolidinediones	0/16 (0.0%)	0/33 (0.0%)	0/8 (0.0%)	0/13 (0.0%)	0/70 (0.0%)
Insulin	9/16 (56.3%)	0/33 (0.0%)	1/8 (12.5%)	0/13 (0.0%)	10/70 (14.3%)
Other	4/4 (100.0%)	5/6 (83.3%)	2/2 (100.0%)	4/6 (66.7%)	15/18 (83.3%)
**Medications at day 28**					
Aspirin	10/10 (100.0%)	28/28 (100.0%)	5/5 (100.0%)	8/8 (100.0%)	51/51 (100.0%)
Clopidogrel	4/10 (40.0%)	11/28 (39.3%)	2/5 (40.0%)	4/8 (50.0%)	21/51 (41.2%)
Prasugrel	6/7 (85.7%)	16/18 (88.9%)	0/2 (0.0%)	2/5 (40.0%)	24/32 (75.0%)
Warfarin	0/10 (0.0%)	1/28 (3.6%)	1/5 (20.0%)	1/8 (12.5%)	3/51 (5.9%)
Heparin	0/10 (0.0%)	0/28 (0.0%)	0/5 (0.0%)	0/8 (0.0%)	0/51 (0.0%)
Clexane	0/10 (0.0%)	0/28 (0.0%)	0/5 (0.0%)	0/8 (0.0%)	0/51 (0.0%)

Medication	STEMI with DM (n = 16)	STEMI without DM (n = 34)	NSTEMI with DM (n = 8)	NSTEMI without DM (n = 13)	Overall (n = 71)
Beta-blockers	7/10 (70.0%)	27/28 (96.4%)	5/5 (100.0%)	7/8 (87.5%)	46/51 (90.2%)
Calcium channel antagonists	3/10 (30.0%)	2/28 (7.1%)	0/5 (0.0%)	0/8 (0.0%)	5/51 (9.8%)
Nitrates	1/10 (10.0%)	2/28 (7.1%)	0/5 (0.0%)	1/8 (12.5%)	4/51 (7.8%)
Potassium channel activators	0/10 (0.0%)	0/28 (0.0%)	0/5 (0.0%)	0/8 (0.0%)	0/51 (0.0%)
Lipid lowering agent	10/10 (100.0%)	27/28 (96.4%)	5/5 (100.0%)	8/8 (100.0%)	50/51 (98.0%)
ACE inhibitors	6/10 (60.0%)	25/28 (89.3%)	4/5 (80.0%)	8/8 (100.0%)	43/51 (84.3%)
Angiotensin-|| antagonists	4/10 (40.0%)	0/28 (0.0%)	1/5 (20.0%)	0/8 (0.0%)	5/51 (9.8%)
Loop diuretics	1/10 (10.0%)	0/28 (0.0%)	2/5 (40.0%)	0/8 (0.0%)	3/51 (5.9%)
Thiazide diuretics	0/10 (0.0%)	0/28 (0.0%)	0/5 (0.0%)	0/8 (0.0%)	0/51 (0.0%)
Digoxin	0/10 (0.0%)	0/28 (0.0%)	0/5 (0.0%)	0/8 (0.0%)	0/51 (0.0%)
Amiodrone	0/10 (0.0%)	0/28 (0.0%)	0/5 (0.0%)	0/8 (0.0%)	0/51 (0.0%)
Thyroxine	0/10 (0.0%)	0/28 (0.0%)	0/5 (0.0%)	0/8 (0.0%)	0/51 (0.0%)
Metformin	6/10 (60.0%)	0/28 (0.0%)	1/5 (20.0%)	0/8 (0.0%)	7/51 (13.7%)
Sulphonylureas	2/10 (20.0%)	1/28 (3.6%)	1/5 (20.0%)	0/8 (0.0%)	4/51 (7.8%)
Thiazolidinediones	0/10 (0.0%)	0/28 (0.0%)	0/5 (0.0%)	0/8 (0.0%)	0/51 (0.0%)
Insulin	6/10 (60.0%)	0/28 (0.0%)	0/5 (0.0%)	0/8 (0.0%)	6/51 (11.8%)
Other	4/4 (100.0%)	6/6 (100.0%)	2/2 (100.0%)	6/7 (85.7%)	18/19 (94.7%)
**Medications at 3 months**					
Aspirin	11/11 (100.0%)	25/25 (100.0%)	4/5 (80.0%)	8/8 (100.0%)	48/49 (98.0%)
Clopidogrel	4/11 (36.4%)	9/25 (36.0%)	3/5 (60.0%)	6/9 (66.7%)	22/50 (44.0%)
Prasugrel	7/7 (100.0%)	14/15 (93.3%)	0/2 (0.0%)	2/4 (50.0%)	23/28 (82.1%)
Warfarin	0/11 (0.0%)	1/25 (4.0%)	1/5 (20.0%)	0/9 (0.0%)	2/50 (4.0%)
Heparin	0/11 (0.0%)	0/25 (0.0%)	0/5 (0.0%)	0/9 (0.0%)	0/50 (0.0%)
Clexane	0/11 (0.0%)	0/25 (0.0%)	1/5 (20.0%)	1/9 (11.1%)	2/50 (4.0%)
Beta-blockers	10/11 (90.9%)	24/25 (96.0%)	5/5 (100.0%)	7/8 (87.5%)	46/49 (93.9%)
Calcium channel antagonists	2/11 (18.2%)	2/25 (8.0%)	0/5 (0.0%)	0/9 (0.0%)	4/50 (8.0%)
Nitrates	0/11 (0.0%)	3/25 (12.0%)	0/5 (0.0%)	0/9 (0.0%)	3/50 (6.0%)
Potassium channel activators	0/11 (0.0%)	0/25 (0.0%)	0/5 (0.0%)	0/9 (0.0%)	0/50 (0.0%)
Lipid lowering agent	9/11 (81.8%)	21/25 (84.0%)	5/5 (100.0%)	9/9 (100.0%)	44/50 (88.0%)
ACE inhibitors	7/11 (63.6%)	23/25 (92.0%)	5/5 (100.0%)	8/9 (88.9%)	43/50 (86.0%)
Angiotensin-|| antagonists	2/11 (18.2%)	1/25 (4.0%)	1/5 (20.0%)	1/9 (11.1%)	5/50 (10.0%)
Loop diuretics	1/11 (9.1%)	0/25 (0.0%)	1/5 (20.0%)	0/9 (0.0%)	2/50 (4.0%)

Medication	STEMI with DM (n = 16)	STEMI without DM (n = 34)	NSTEMI with DM (n = 8)	NSTEMI without DM (n = 13)	Overall (n = 71)
Thiazide diuretics	1/11 (9.1%)	0/25 (0.0%)	1/5 (20.0%)	0/9 (0.0%)	2/50 (4.0%)
Digoxin	0/11 (0.0%)	0/25 (0.0%)	0/5 (0.0%)	0/9 (0.0%)	0/50 (0.0%)
Amiodrone	1/11 (9.1%)	0/25 (0.0%)	0/5 (0.0%)	0/9 (0.0%)	1/50 (2.0%)
Thyroxine	1/11 (9.1%)	0/25 (0.0%)	0/5 (0.0%)	0/9 (0.0%)	1/50 (2.0%)
Metformin	5/11 (45.5%)	0/25 (0.0%)	1/5 (20.0%)	0/9 (0.0%)	6/50 (12.0%)
Sulphonylureas	2/11 (18.2%)	0/25 (0.0%)	1/5 (20.0%)	0/9 (0.0%)	3/50 (6.0%)
Thiazolidinediones	0/11 (0.0%)	0/25 (0.0%)	1/5 (20.0%)	0/9 (0.0%)	1/50 (2.0%)
Insulin	5/11 (45.5%)	0/25 (0.0%)	0/5 (0.0%)	0/9 (0.0%)	5/50 (10.0%)
Other	1/1 (100.0%)	5/5 (100.0%)	2/2 (100.0%)	5/5 (100.0%)	13/13 (100.0%)
**Medications at 1 year**					
Aspirin	15/15 (100.0%)	30/32 (93.8%)	6/7 (85.7%)	9/11 (81.8%)	60/65 (92.3%)
Clopidogrel	3/15 (20.0%)	5/32 (15.6%)	2/7 (28.6%)	8/11 (72.7%)	18/65 (27.7%)
Prasugrel	6/11 (54.5%)	14/19 (73.7%)	0/3 (0.0%)	2/6 (33.3%)	22/39 (56.4%)
Warfarin	1/15 (6.7%)	2/32 (6.3%)	1/7 (14.3%)	0/11 (0.0%)	4/65 (6.2%)
Heparin	0/15 (0.0%)	0/32 (0.0%)	0/7 (0.0%)	0/11 (0.0%)	0/65 (0.0%)
Clexane	0/15 (0.0%)	0/32 (0.0%)	0/7 (0.0%)	0/11 (0.0%)	0/65 (0.0%)
Beta-blockers	13/15 (86.7%)	30/32 (93.8%)	7/7 (100.0%)	10/11 (90.9%)	60/65 (92.3%)
Calcium channel antagonists	2/15 (13.3%)	1/32 (3.1%)	0/7 (0.0%)	0/11 (0.0%)	3/65 (4.6%)
Nitrates	2/15 (13.3%)	2/32 (6.3%)	0/7 (0.0%)	1/11 (9.1%)	5/65 (7.7%)
Potassium channel activators	0/15 (0.0%)	0/32 (0.0%)	0/7 (0.0%)	0/11 (0.0%)	0/65 (0.0%)
Lipid lowering agent	13/15 (86.7%)	27/32 (84.4%)	6/7 (85.7%)	10/11 (90.9%)	56/65 (86.2%)
ACE inhibitors	10/15 (66.7%)	29/32 (90.6%)	6/7 (85.7%)	9/11 (81.8%)	54/65 (83.1%)
Angiotensin-|| antagonists	5/15 (33.3%)	2/32 (6.3%)	0/7 (0.0%)	1/11 (9.1%)	8/65 (12.3%)
Loop diuretics	2/15 (13.3%)	0/32 (0.0%)	2/7 (28.6%)	0/11 (0.0%)	4/65 (6.2%)
Thiazide diuretics	1/15 (6.7%)	2/32 (6.3%)	1/7 (14.3%)	0/11 (0.0%)	4/65 (6.2%)
Digoxin	0/15 (0.0%)	0/32 (0.0%)	1/7 (14.3%)	0/11 (0.0%)	1/65 (1.5%)
Amiodrone	0/15 (0.0%)	0/32 (0.0%)	0/7 (0.0%)	0/11 (0.0%)	0/65 (0.0%)
Thyroxine	2/15 (13.3%)	0/32 (0.0%)	0/7 (0.0%)	0/11 (0.0%)	2/65 (3.1%)
Metformin	7/15 (46.7%)	1/32 (3.1%)	4/7 (57.1%)	0/11 (0.0%)	12/65 (18.5%)
Sulphonylureas	3/15 (20.0%)	1/32 (3.1%)	4/7 (57.1%)	0/11 (0.0%)	8/65 (12.3%)
Thiazolidinediones	0/15 (0.0%)	0/32 (0.0%)	1/7 (14.3%)	0/11 (0.0%)	1/65 (1.5%)
Insulin	7/15 (46.7%)	0/32 (0.0%)	0/7 (0.0%)	0/11 (0.0%)	7/65 (10.8%)

Medication	STEMI with DM (n = 16)	STEMI without DM (n = 34)	NSTEMI with DM (n = 8)	NSTEMI without DM (n = 13)	Overall (n = 71)
Other	6/9 (66.7%)	9/12 (75.0%)	3/3 (100.0%)	4/6 (66.7%)	22/30 (73.3%)

Notes:

Data are presented as n (%).

Denominators are the number of patients with non-missing data.

Abbreviations: *DM=Diabetes Mellitus, STEMI=ST-elevation myocardial infarction, NSTEMI=Non-ST-elevation myocardial infarction*

Supplementary Table 6: CPC lab data and MRI data available

CPC data/MRI data available	STEMI with DM (n = 16)	STEMI without DM (n = 34)	NSTEMI with DM (n = 8)	NSTEMI without DM (n = 13)	Overall (n = 71)
**MRI data**					
No MRI	2 (12.5%)	4 (11.8%)	0 (0.0%)	2 (15.4%)	8 (11.3%)
MRI baseline only	3 (18.8%)	3 (8.8%)	1 (12.5%)	1 (7.7%)	8 (11.3%)
MRI 3 month only	0 (0.0%)	1 (2.9%)	2 (25.0%)	0 (0.0%)	3 (4.2%)
MRI baseline and 3 months	11 (68.8%)	26 (76.5%)	5 (62.5%)	10 (76.9%)	52 (73.2%)
**CPC data**					
No CPC data	2 (12.5%)	3 (8.8%)	2 (25.0%)	6 (46.2%)	13 (18.3%)
CPC day 0 only*	0 (0.0%)	2 (5.9%)	0 (0.0%)	1 (7.7%)	3 (4.2%)
CPC day 4 only*	0 (0.0%)	0 (0.0%)	0 (0.0%)	0 (0.0%)	0 (0%)
CPC day 0 and day 4*	14 (87.5%)	29 (85.3%)	6 (75.0%)	6 (46.2%)	55 (77.5%)
**Migratory CPC data (day 4 only)**					
No migratory CPC data	2 (12.5%)	7 (20.6%)	3 (37.5%)	7 (53.9%)	19 (26.8%)
Migratory CPC data**	14 (87.5%)	27 (79.4%)	5 (62.5%)	6 (46.2%)	52 (73.2%)
**CPC and MRI data**					
No CPC or MRI data	1 (6.3%)	1 (2.9%)	0 (0.0%)	0 (0.0%)	2 (2.8%)
Missing CPC on day 0 and day 4 or MRI at baseline or 3 months	4 (25.0%)	9 (26.5%)	5 (62.5%)	8 (61.5%)	26 (36.6%)
CPC day 0 and/or 4 and MRI at baseline and 3 months***	11 (68.8%)	24 (70.6%)	3 (37.5%)	5 (38.5%)	43 (60.6%)
**Migratory CPC data and MRI data**					
No migratory CPC or MRI data	1 (6.3%)	2 (5.9%)	0 (0.0%)	1 (7.7%)	4 (5.6%)
Missing migratory CPC data or MRI at baseline or 3 months	4 (25.0%)	11 (32.3%)	5 (62.5%)	7 (53.8%)	27 (38.0%)
Migratory CPC data and MRI at baseline and 3 months****	11 (68.8%)	21 (61.8%)	3 (37.5%)	5 (38.5%)	40 (56.3%)

** Analysis population for objective 1a (n=58)*

*** Analysis population for objective 1b (n=52)*** Analysis population for objective 2a (n=43)*

***** Analysis population for objective 2b (n=40)*

Supplementary Table 7: CPC measurements

		STEMI with DM (n = 16)	STEMI without DM (n = 34)	NSTEMI with DM (n = 8)	NSTEMI without DM (n = 13)	Overall (n = 71*)
**Percentage of cells**						
KDR+	Day 0	0.027 (0.019, 0.034)	0.025 (0.012, 0.044)	0.032 (0.022, 0.038)	0.014 (0.005, 0.018)	0.025 (0.014, 0.034)
	Day 4	0.020 (0.012, 0.049)	0.019 (0.012, 0.036)	0.034 (0.020, 0.040)	0.027 (0.023, 0.034)	0.021 (0.012, 0.040)
CD133+	Day 0	0.035 (0.020, 0.077)	0.043 (0.016, 0.068)	0.046 (0.032, 0.053)	0.036 (0.008, 0.069)	0.040 (0.020, 0.067)
	Day 4	0.028 (0.010, 0.049)	0.069 (0.039, 0.091)	0.067 (0.022, 0.117)	0.050 (0.008, 0.069)	0.050 (0.022, 0.084)
CD34+	Day 0	0.080 (0.041, 0.150)	0.096 (0.064, 0.197)	0.065 (0.032, 0.097)	0.114 (0.051, 0.134)	0.091 (0.053, 0.173)
	Day 4	0.141 (0.080, 0.166)	0.139 (0.104, 0.236)	0.200 (0.095, 0.257)	0.110 (0.077, 0.130)	0.139 (0.091, 0.228)
CD34+/CD133	Day 0	0.011 (0.001, 0.020)	0.018 (0.006, 0.063)	0.025 (0.015, 0.038)	0.039 (0.000, 0.084)	0.017 (0.005, 0.053)
	Day 4	0.008 (0.001, 0.023)	0.032 (0.011, 0.052)	0.023 (0.016, 0.068)	0.024 (0.001, 0.069)	0.023 (0.006, 0.051)
CD34+/KDR+	Day 0	0.001 (0.000, 0.003)	0.002 (0.001, 0.007)	0.002 (0.000, 0.003)	0.002 (0.001, 0.003)	0.002 (0.000, 0.004)
	Day 4	0.000 (0.000, 0.002)	0.003 (0.002, 0.006)	0.002 (0.000, 0.004)	0.001 (0.000, 0.003)	0.002 (0.000, 0.005)
CD133+/KDR+	Day 0	0.003 (0.000, 0.008)	0.004 (0.001, 0.015)	0.009 (0.002, 0.011)	0.001 (0.001, 0.003)	0.003 (0.001, 0.009)
	Day 4	0.001 (0.000, 0.002)	0.004 (0.001, 0.009)	0.001 (0.000, 0.003)	0.002 (0.000, 0.003)	0.002 (0.000, 0.006)
CXCR4+	Day 0	12.9 (10.6, 16.6)	8.0 (4.4, 25.8)	14.7 (10.5, 17.5)	11.0 (3.8, 12.3)	11.7 (7.1, 17.5)
	Day 4	7.9 (4.2, 10.6)	9.0 (5.7, 15.1)	16.7 (9.5, 25.4)	8.8 (5.7, 14.5)	9.0 (5.7, 15.1)
CD34+/CXCR4+	Day 0	0.045 (0.030, 0.143)	0.038 (0.020, 0.143)	0.045 (0.026, 0.068)	0.016 (0.012, 0.021)	0.037 (0.020, 0.090)
	Day 4	0.074 (0.028, 0.214)	0.065 (0.022, 0.128)	0.134 (0.015, 0.197)	0.020 (0.017, 0.050)	0.058 (0.017, 0.150)
CD133+/CXCR4+	Day 0	0.006 (0.004, 0.010)	0.015 (0.005, 0.034)	0.008 (0.006, 0.011)	0.007 (0.003, 0.010)	0.008 (0.005, 0.018)
	Day 4	0.003 (0.000, 0.010)	0.014 (0.005, 0.052)	0.012 (0.004, 0.023)	0.007 (0.002, 0.013)	0.010 (0.002, 0.026)
CD164+	Day 0	0.333 (0.101, 0.663)	0.283 (0.078, 0.632)	0.126 (0.038, 0.207)	0.295 (0.107, 0.528)	0.265 (0.093, 0.632)
	Day 4	0.254 (0.118, 0.353)	0.274 (0.152, 0.579)	0.205 (0.146, 0.545)	0.450 (0.264, 1.168)	0.274 (0.146, 0.529)
CD34+/CD164+	Day 0	0.004 (0.002, 0.015)	0.013 (0.006, 0.036)	0.009 (0.003, 0.010)	0.008 (0.001, 0.012)	0.009 (0.004, 0.025)
	Day 4	0.006 (0.003, 0.017)	0.018 (0.008, 0.062)	0.010 (0.004, 0.027)	0.024 (0.012, 0.032)	0.015 (0.006, 0.042)
CD133+/CD164+	Day 0	0.004 (0.002, 0.008)	0.006 (0.001, 0.030)	0.002 (0.000, 0.006)	0.002 (0.001, 0.006)	0.004 (0.001, 0.009)
	Day 4	0.001 (0.000, 0.002)	0.006 (0.003, 0.024)	0.002 (0.000, 0.008)	0.006 (0.002, 0.007)	0.004 (0.001, 0.013)
C-KIT+	Day 0	0.539 (0.260, 0.810)	0.470 (0.262, 1.016)	0.384 (0.328, 0.723)	0.857 (0.616, 1.092)	0.474 (0.324, 0.907)
	Day 4	0.471 (0.267, 0.731)	0.515 (0.335, 0.817)	0.673 (0.573, 0.822)	0.908 (0.500, 1.133)	0.550 (0.314, 0.822)
CD34+/C-KIT+	Day 0	0.028 (0.016, 0.063)	0.049 (0.032, 0.120)	0.030 (0.022, 0.046)	0.050 (0.033, 0.101)	0.045 (0.028, 0.081)
	Day 4	0.034 (0.012, 0.054)	0.069 (0.035, 0.119)	0.045 (0.022, 0.077)	0.066 (0.030, 0.092)	0.056 (0.029, 0.105)

		STEMI with DM (n = 16)	STEMI without DM (n = 34)	NSTEMI with DM (n = 8)	NSTEMI without DM (n = 13)	Overall (n = 71*)
CD133+/C-KIT+	Day 0	0.017 (0.006, 0.035)	0.033 (0.010, 0.046)	0.021 (0.013, 0.022)	0.022 (0.001, 0.058)	0.021 (0.006, 0.046)
	Day 4	0.007 (0.000, 0.024)	0.043 (0.013, 0.064)	0.019 (0.011, 0.028)	0.017 (0.003, 0.058)	0.022 (0.010, 0.048)
TRKA+	Day 0	0.7 (0.3, 0.9)	0.7 (0.4, 1.8)	1.0 (0.7, 1.4)	0.8 (0.4, 3.0)	0.7 (0.4, 1.3)
	Day 4	0.9 (0.4, 1.3)	0.9 (0.5, 2.2)	1.5 (0.9, 2.0)	1.6 (1.0, 2.5)	1.0 (0.6, 2.0)
CD34+/TRKA+	Day 0	0.008 (0.004, 0.017)	0.018 (0.007, 0.036)	0.007 (0.004, 0.011)	0.007 (0.005, 0.008)	0.012 (0.005, 0.030)
	Day 4	0.012 (0.008, 0.038)	0.031 (0.008, 0.071)	0.009 (0.005, 0.011)	0.013 (0.006, 0.014)	0.014 (0.007, 0.059)
CD133+/TRKA+	Day 0	0.003 (0.002, 0.006)	0.004 (0.001, 0.013)	0.003 (0.000, 0.007)	0.001 (0.000, 0.004)	0.003 (0.001, 0.009)
	Day 4	0.002 (0.000, 0.007)	0.006 (0.000, 0.038)	0.003 (0.001, 0.006)	0.003 (0.002, 0.003)	0.003 (0.001, 0.015)
CD14+	Day 0	83.8 (79.1, 87.4)	87.1 (76.3, 90.7)	90.6 (87.1, 93.2)	88.7 (83.6, 91.6)	86.7 (79.1, 91.6)
	Day 4	82.5 (68.0, 88.2)	83.8 (77.0, 91.1)	83.3 (77.9, 84.8)	81.6 (74.5, 86.9)	82.8 (76.4, 88.2)
CD16+	Day 0	21.4 (15.2, 29.6)	13.9 (10.0, 18.6)	17.0 (11.1, 26.7)	15.4 (8.2, 18.1)	15.5 (11.1, 25.5)
	Day 4	17.1 (12.8, 26.8)	16.7 (10.8, 27.6)	17.6 (14.0, 23.5)	24.3 (19.2, 26.4)	17.7 (10.8, 26.9)
CD14+/CD16+	Day 0	7.2 (5.4, 12.3)	6.0 (3.4, 12.6)	11.5 (5.0, 20.1)	3.6 (2.6, 5.7)	5.9 (3.5, 12.3)
	Day 4	7.8 (2.8, 10.9)	6.1 (3.5, 12.7)	6.6 (5.4, 7.2)	6.8 (2.9, 9.2)	7.0 (3.3, 10.9)
CD14+/CD16-	Day 0	71.6 (64.4, 78.6)	80.0 (55.2, 84.3)	75.6 (70.3, 84.0)	82.0 (79.2, 86.5)	78.3 (61.5, 83.2)
	Day 4	74.7 (52.0, 80.0)	73.4 (65.0, 82.6)	72.8 (67.5, 76.4)	72.3 (66.4, 75.5)	73.6 (65.0, 80.2)

Notes:

Data are presented as mean (SD) or median (IQR)

* *Samples analysed for 58 patients (14, 31, 6, 7) at day 0 and 55 patients at day 4 (14, 29, 6, 6).*

For each patient, the mean of replicate measurements is summarised.

Abbreviations: *DM=Diabetes Mellitus, STEMI=ST-elevation myocardial infarction, NSTEMI=Non-ST-elevation myocardial infarction*

Supplementary Table 8: Migrated cells on day 4

		STEMI with DM (n = 14)	STEMI without DM (n = 27)	NSTEMI with DM (n = 5)	NSTEMI without DM (n = 6)	Overall (n = 52)
**Percentages of migrated cells**						
KDR+	Vehicle	0.065 (0.028, 0.221)	0.050 (0.022, 0.384)	0.391 (0.212, 0.566)	0.039 (0.028, 0.267)	0.078 (0.028, 0.365)
	SDF	0.058 (0.042, 0.129)	0.043 (0.025, 0.348)	0.153 (0.133, 0.344)	0.029 (0.027, 0.113)	0.054 (0.027, 0.278)
	NGF	0.060 (0.047, 0.113)	0.049 (0.025, 0.247)	0.201 (0.156, 0.505)	0.028 (0.023, 0.140)	0.060 (0.027, 0.196)
CD133+	Vehicle	0.040 (0.024, 0.415)	0.120 (0.026, 0.390)	0.075 (0.061, 0.101)	0.151 (0.080, 0.261)	0.085 (0.027, 0.353)
	SDF	0.045 (0.028, 0.229)	0.108 (0.037, 0.271)	0.070 (0.060, 0.093)	0.122 (0.080, 0.337)	0.079 (0.033, 0.264)
	NGF	0.043 (0.027, 0.316)	0.107 (0.026, 0.185)	0.070 (0.048, 0.078)	0.101 (0.060, 0.221)	0.078 (0.027, 0.227)
CD34+	Vehicle	0.072 (0.046, 0.184)	0.190 (0.063, 0.315)	0.118 (0.080, 0.148)	0.196 (0.015, 0.413)	0.144 (0.060, 0.283)
	SDF	0.091 (0.049, 0.146)	0.180 (0.085, 0.368)	0.095 (0.092, 0.133)	0.198 (0.031, 0.318)	0.125 (0.068, 0.261)
	NGF	0.072 (0.044, 0.129)	0.148 (0.082, 0.338)	0.095 (0.062, 0.179)	0.146 (0.023, 0.243)	0.110 (0.065, 0.267)
CD34+/CD133+	Vehicle	0.006 (0.002, 0.040)	0.042 (0.013, 0.081)	0.049 (0.024, 0.100)	0.040 (0.010, 0.112)	0.033 (0.008, 0.069)
	SDF	0.011 (0.003, 0.043)	0.043 (0.018, 0.112)	0.027 (0.009, 0.091)	0.049 (0.021, 0.106)	0.037 (0.008, 0.091)
	NGF	0.007 (0.002, 0.022)	0.044 (0.023, 0.097)	0.020 (0.018, 0.085)	0.030 (0.017, 0.056)	0.026 (0.008, 0.065)
CD34+/KDR+	Vehicle	0.000 (0.000, 0.004)	0.005 (0.001, 0.030)	0.008 (0.008, 0.025)	0.005 (0.003, 0.007)	0.005 (0.001, 0.014)
	SDF	0.001 (0.000, 0.001)	0.004 (0.001, 0.018)	0.008 (0.008, 0.020)	0.006 (0.003, 0.011)	0.003 (0.001, 0.010)
	NGF	0.000 (0.000, 0.005)	0.005 (0.002, 0.027)	0.019 (0.007, 0.021)	0.004 (0.003, 0.007)	0.005 (0.001, 0.013)
CD133+/KDR+	Vehicle	0.004 (0.000, 0.013)	0.008 (0.002, 0.047)	0.012 (0.003, 0.070)	0.010 (0.004, 0.018)	0.008 (0.002, 0.022)
	SDF	0.003 (0.001, 0.004)	0.007 (0.002, 0.028)	0.005 (0.004, 0.053)	0.003 (0.002, 0.009)	0.004 (0.001, 0.017)
	NGF	0.005 (0.002, 0.010)	0.006 (0.002, 0.038)	0.007 (0.005, 0.066)	0.005 (0.003, 0.008)	0.006 (0.002, 0.016)
CXCR4+	Vehicle	61.3 (15.0, 75.5)	57.5 (25.8, 76.8)	56.8 (44.0, 61.6)	58.2 (34.0, 62.9)	58.4 (25.9, 69.6)
	SDF	60.6 (23.5, 83.9)	60.7 (27.1, 85.7)	62.8 (55.2, 85.0)	35.7 (28.4, 53.1)	56.3 (28.4, 83.1)
	NGF	72.3 (30.9, 78.8)	60.3 (40.5, 83.5)	59.9 (58.2, 72.4)	65.5 (50.9, 67.7)	64.1 (41.3, 77.8)
CD34+/CXCR4+	Vehicle	0.045 (0.010, 0.105)	0.117 (0.049, 0.273)	0.103 (0.066, 0.164)	0.040 (0.026, 0.179)	0.087 (0.031, 0.186)
	SDF	0.028 (0.011, 0.068)	0.099 (0.037, 0.283)	0.084 (0.083, 0.092)	0.026 (0.017, 0.068)	0.068 (0.026, 0.135)
	NGF	0.052 (0.015, 0.064)	0.119 (0.042, 0.270)	0.070 (0.048, 0.137)	0.044 (0.022, 0.130)	0.063 (0.031, 0.192)
CD133+/CXCR4+	Vehicle	0.018 (0.005, 0.059)	0.077 (0.018, 0.206)	0.103 (0.073, 0.145)	0.036 (0.021, 0.043)	0.042 (0.013, 0.121)
	SDF	0.008 (0.004, 0.038)	0.059 (0.019, 0.178)	0.080 (0.049, 0.082)	0.015 (0.013, 0.023)	0.034 (0.011, 0.081)
	NGF	0.025 (0.010, 0.061)	0.069 (0.025, 0.181)	0.081 (0.067, 0.084)	0.026 (0.018, 0.041)	0.046 (0.018, 0.101)

Notes: Data are presented as median (IQR). * Samples analysed for 52 patients (14, 27, 5, 6) For each patient, the mean of replicate measurements is summarised. **Abbreviations:** DM=Diabetes Mellitus, STEMI=ST-elevation myocardial infarction, NSTEMI=Non-ST-elevation myocardial infarction

Supplementary Table 9: Cardiac MRI at baseline

	STEMI with DM (n = 14)	STEMI without DM (n = 29)	NSTEMI with DM (n = 6)	NSTEMI without DM (n = 11)	Overall (n = 60)
**LV absolute**					
Ejection Fraction (%)	54.8 (13.3)	54.3 (9.5)	53.5 (12.6)	55.4 (8.0)	54.0 (11.0)
End Diastolic Volume (ml/m^2)	138.7 (31.5)	154.0 (30.9)	135.7 (24.9)	160.5 (24.6)	150.7 (30.7)
End Systolic Volume (ml/m^2)	65.0 (28.9)	71.8 (24.1)	64.8 (26.4)	72.6 (20.5)	71.1 (26.9)
Stroke Volume (ml)	73.6 (15.1)	84.7 (16.6)	70.5 (13.8)	87.9 (13.0)	80.7 (16.8)
Mass	125.2 (23.7)	142.5 (34.0)	126.0 (13.8)	138.6 (23.8)	137.1 (29.7)
**LV indexed**					
End Diastolic Volume (ml/m^2)	69.3 (13.7)	77.8 (16.0)	65.8 (7.5)	80.1 (12.8)	75.5 (15.1)
End Systolic Volume (ml/m^2)	32.2 (13.2)	36.5 (13.4)	31.3 (11.5)	36.1 (9.3)	35.6 (13.4)
Stroke Volume (ml)	37.1 (7.9)	42.4 (6.3)	34.2 (4.9)	44.1 (8.1)	40.3 (7.8)
Mass	62.5 (9.5)	71.6 (15.7)	61.6 (6.2)	69.2 (12.1)	68.5 (13.8)
Longitudinal strain (%)[a]	-16.2 (4.5)	-16.6 (4.1)	-18.2 (6.2)	-17.2 (2.6)	-16.8 (4.1)
Infarct size (g) [b]	12.5 (6.0, 22.0)	15.0 (8.5, 19.0)	11.0 (3.0, 25.0)	12.0 (3.0, 21.0)	12.5 (6.5, 20.5)
Infarct size (%) [c]	17.6 (11.3, 31.4)	20.9 (11.5, 31.9)	17.6 (4.8, 40.2)	14.6 (5.5, 28.5)	18.3 (9.8, 31.2)
Oedema (g) [d]	16.0 (2.0, 38.0)	19.0 (7.0, 28.0)	19.0 (0.0, 27.0)	7.0 (0.0, 26.0)	15.5 (2.0, 27.0)
Oedema (%) [e]	23.4 (3.8, 54.3)	27.9 (9.7, 40.1)	30.2 (0.0, 43.4)	9.2 (0.0, 28.9)	22.6 (3.8, 43.9)
Feature tracking	14/14 (100.0%)	26/29 (89.7%)	6/6 (100.0%)	11/11 (100.0%)	57/61 (93.4%)

Notes:

Data are presented as median (IQR), mean (SD) or n (%)

Missing data (STEMI with DM, STEMI without DM, NSTEMI with DM, NSTEMI without DM):

[a] Data missing for 3 patients (0, 3, 0, 0)

[b] Data missing for 1 patients (0, 1, 0, 0)

[c] Data missing for 1 patients (0, 1, 0, 0)

[d] Data missing for 3 patients (0, 3, 0, 0)

[e] Data missing for 3 patients (0, 3, 0, 0)

***Abbreviations:** DM=Diabetes Mellitus, STEMI=ST-elevation myocardial infarction, NSTEMI=Non-ST-elevation myocardial infarction, LV=Left ventricular*

Supplementary Table 10: Cardiac MRI at 3 months

	STEMI with DM (n = 11)	STEMI without DM (n = 27)	NSTEMI with DM (n = 7)	NSTEMI without DM (n = 10)	Overall (n = 55)
**LV absolute**					
Ejection Fraction (%)	58.5 (11.3)	58.7 (8.0)	52.1 (12.7)	60.0 (5.2)	58.0 (9.1)
End Diastolic Volume (ml/m^2)	132.8 (28.9)	158.7 (34.7)	153.9 (41.7)	153.6 (26.0)	152.0 (33.7)
End Systolic Volume (ml/m^2)	56.8 (25.0)	67.2 (24.4)	75.7 (37.9)	60.7 (7.8)	65.0 (24.7)
Stroke Volume (ml)	75.9 (12.6)	91.3 (16.4)	78.3 (21.9)	93.9 (20.7)	87.0 (18.3)
Mass	120.7 (30.0)	133.6 (30.6)	128.0 (23.8)	132.6 (19.2)	130.1 (27.7)
**LV indexed**					
End Diastolic Volume (ml/m^2)	65.7 (12.7)	78.5 (16.9)	77.7 (24.1)	77.4 (14.3)	75.7 (17.1)
End Systolic Volume (ml/m^2)	28.0 (11.8)	33.4 (12.9)	38.4 (21.4)	30.6 (4.4)	32.4 (13.0)
Stroke Volume (ml)	37.7 (6.2)	45.1 (6.6)	39.4 (11.1)	47.3 (11.2)	43.3 (8.7)
Mass	59.2 (10.3)	65.6 (11.7)	64.3 (13.0)	66.7 (9.8)	64.4 (11.3)
Longitudinal strain (%)[a]	-18.9 (4.0)	-19.1 (3.3)	-16.4 (4.6)	-18.2 (3.1)	-18.5 (3.6)
Infarct size (g) [b]	9.0 (4.0, 17.0)	7.0 (5.0, 14.0)	9.0 (3.0, 18.0)	4.5 (1.0, 22.0)	8.0 (4.0, 17.0)
Infarct size (%) [c]	12.1 (6.2, 31.3)	11.8 (6.6, 24.5)	16.5 (4.7, 33.2)	5.7 (1.8, 30.9)	12.0 (5.6, 26.8)
Oedema (g) [d]	0.0 (0.0, 0.0)	0.0 (0.0, 0.0)	0.0 (0.0, 6.0)	0.0 (0.0, 0.0)	0.0 (0.0, 0.0)
Oedema (%) [e]	0.0 (0.0, 0.0)	0.0 (0.0, 0.0)	0.0 (0.0, 9.4)	0.0 (0.0, 0.0)	0.0 (0.0, 0.0)
Feature tracking	11/11 (100.0%)	26/27 (96.3%)	7/7 (100.0%)	10/10 (100.0%)	54/55 (98.2%)

Notes:
Data are presented as median (IQR), mean (SD) or n (%)
Missing data (STEMI with DM, STEMI without DM, NSTEMI with DM, NSTEMI without DM):
[a] Data missing for 1 patient (0, 1, 0, 0)
[b] Data missing for 2 patients (0, 2, 0, 0)
[c] Data missing for 2 patients (0, 2, 0, 0)
[d] Data missing for 1 patient (0, 1, 0, 0)
[e] Data missing for 1 patient (0, 1, 0, 0)
Abbreviations: *DM=Diabetes Mellitus, STEMI=ST-elevation myocardial infarction, NSTEMI=Non-ST-elevation myocardial infarction, LV=Left ventricular*

Supplementary Table 11: Expected adverse events and serious adverse events

	STEMI with DM (n = 16)	STEMI without DM (n = 34)	NSTEMI with DM (n = 8)	NSTEMI without DM (n = 13)	Overall (n = 71)
**Expected events**					
**Perioperative**					
Death	0/16 (0.0%)	0/33 (0.0%)	0/8 (0.0%)	0/13 (0.0%)	0/70 (0.0%)
Myocardial infarction	0/16 (0.0%)	0/33 (0.0%)	0/8 (0.0%)	0/13 (0.0%)	0/70 (0.0%)
Cerebrovascular event	0/16 (0.0%)	0/33 (0.0%)	0/8 (0.0%)	0/13 (0.0%)	0/70 (0.0%)
Major/minor bleed	0/16 (0.0%)	0/33 (0.0%)	0/8 (0.0%)	2/13 (15.4%)	2/70 (2.9%)
SAE	-	-	-	0/1 (0.0%)	0/1 (0.0%)
Related	-	-	-	-	-
Revascularisation	0/16 (0.0%)	0/33 (0.0%)	1/8 (12.5%)	1/13 (7.7%)	2/70 (2.9%)
SAE	-	-	1/1 (100.0%)	-	1/1 (100.0%)
Related	-	-	1/1 (100.0%)	-	1/1 (100.0%)
Recurrent angina	0/5 (0.0%)	0/6 (0.0%)	0/3 (0.0%)	0/6 (0.0%)	0/20 (0.0%)
Other SAE*	0/16 (0.0%)	1/34 (2.9%)	0/8 (0.0%)	1/13 (7.7%)	2/71 (2.8%)
SAE	-	1/1 (100.0%)	-	1/1 (100.0%)	2/2 (100.0%)
Related	-	1/1 (100.0%)	-	1/1 (100.0%)	2/2 (100.0%)
**28 days**					
Death	0/10 (0.0%)	0/28 (0.0%)	0/5 (0.0%)	0/8 (0.0%)	0/51 (0.0%)
Myocardial infarction	1/10 (10.0%)	0/28 (0.0%)	0/5 (0.0%)	0/8 (0.0%)	1/51 (2.0%)
SAE	1/1 (100.0%)	-	-	-	1/1 (100.0%)
Related	1/1 (100.0%)	-	-	-	1/1 (100.0%)
Cerebrovascular event	0/10 (0.0%)	0/28 (0.0%)	0/5 (0.0%)	0/8 (0.0%)	0/51 (0.0%)
Major/minor bleed	0/10 (0.0%)	0/28 (0.0%)	0/5 (0.0%)	0/8 (0.0%)	0/51 (0.0%)
Revascularisation	1/10 (10.0%)	1/28 (3.6%)	0/5 (0.0%)	0/8 (0.0%)	2/51 (3.9%)
SAE	1/1 (100.0%)	1/1 (100.0%)	-	-	2/2 (100.0%)
Related	1/1 (100.0%)	1/1 (100.0%)	-	-	2/2 (100.0%)
Recurrent angina	0/4 (0.0%)	0/6 (0.0%)	0/3 (0.0%)	0/6 (0.0%)	0/19 (0.0%)
Other SAE/Re-admission**	2/16 (12.5%)	3/34 (8.8%)	1/8 (12.5%)	1/13 (7.7%)	7/71 (9.9%)
SAE	1/1 (100.0%)	1/1 (100.0%)	-	1/1 (100.0%)	3/3 (100.0%)
Related	1/1 (100.0%)	1/1 (100.0%)	-	1/1 (100.0%)	3/3 (100.0%)
**3 months**					
Death	0/11 (0.0%)	0/25 (0.0%)	0/5 (0.0%)	0/9 (0.0%)	0/50 (0.0%)

	STEMI with DM (n = 16)	STEMI without DM (n = 34)	NSTEMI with DM (n = 8)	NSTEMI without DM (n = 13)	Overall (n = 71)
Myocardial infarction	0/11 (0.0%)	0/25 (0.0%)	0/5 (0.0%)	0/9 (0.0%)	0/50 (0.0%)
Cerebrovascular event	0/11 (0.0%)	0/25 (0.0%)	0/5 (0.0%)	0/9 (0.0%)	0/50 (0.0%)
Major/minor bleed	1/11 (9.1%)	0/25 (0.0%)	0/5 (0.0%)	0/9 (0.0%)	1/50 (2.0%)
SAE	0/1 (0.0%)	-	-	-	0/1 (0.0%)
Related	-	-	-	-	-
Revascularisation	0/11 (0.0%)	1/25 (4.0%)	0/5 (0.0%)	0/9 (0.0%)	1/50 (2.0%)
SAE		1/1 (100.0%)	-	-	1/1 (100.0%)
Related		1/1 (100.0%)	-	-	1/1 (100.0%)
Recurrent angina	0/2 (0.0%)	0/5 (0.0%)	0/3 (0.0%)	0/4 (0.0%)	0/14 (0.0%)
Other SAE/Re-admission***	1/16 (6.3%)	5/34 (14.7%)	0/8 (0.0%)	1/13 (7.7%)	7/71 (9.9%)
SAE	-	1/2 (50.0%)	-	-	1/2 (50.0%)
Related	-	1/1 (100.0%)	-	-	1/1 (100.0%)
**1 year**					
Death	0/15 (0.0%)	0/32 (0.0%)	0/7 (0.0%)	0/11 (0.0%)	0/65 (0.0%)
Myocardial infarction	1/15 (6.7%)	0/32 (0.0%)	0/7 (0.0%)	2/11 (18.2%)	3/65 (4.6%)
SAE	1/1 (100.0%)	-	-	2/2 (100.0%)	3/3 (100.0%)
Related	1/1 (100.0%)	-	-	2/2 (100.0%)	3/3 (100.0%)
Cerebrovascular event	0/15 (0.0%)	0/32 (0.0%)	0/7 (0.0%)	0/11 (0.0%)	0/65 (0.0%)
Major/minor bleed	0/15 (0.0%)	0/32 (0.0%)	0/7 (0.0%)	0/11 (0.0%)	0/65 (0.0%)
Revascularisation	1/15 (6.7%)	0/32 (0.0%)	0/7 (0.0%)	2/11 (18.2%)	3/65 (4.6%)
SAE	1/1 (100.0%)	-	-	2/2 (100.0%)	3/3 (100.0%)
Related	1/1 (100.0%)	-	-	2/2 (100.0%)	3/3 (100.0%)
Recurrent angina	1/9 (11.1%)	0/12 (0.0%)	0/3 (0.0%)	0/6 (0.0%)	1/30 (3.3%)
SAE	1/1 (100.0%)	-	-	-	1/1 (100.0%)
Related	1/1 (100.0%)	-	-	-	1/1 (100.0%)
Other SAE/Re-admission****	5/16 (31.3%)	4/34 (11.8%)	2/8 (25.0%)	1/13 (7.7%)	12/71 (16.9%)
SAE	3/3 (100.0%)	4/4 (100.0%)	1/1 (100.0%)	1/1 (100.0%)	9/9 (100.0%)
Related	3/3 (100.0%)	4/4 (100.0%)	1/1 (100.0%)	1/1 (100.0%)	9/9 (100.0%)
**Unexpected events** [#]					
At least one unexpected event	4/16 (25.0%)	8/34 (23.5%)	2/8 (25.0%)	2/13 (15.4%)	16/71 (22.5%)

	STEMI with DM (n = 16)	STEMI without DM (n = 34)	NSTEMI with DM (n = 8)	NSTEMI without DM (n = 13)	Overall (n = 71)
Number of events	8	13	3	5	29

Notes:

SAEs are a subset of AEs. Denominators of SAE and relatedness are the number of patients with the event and SAE/relatedness data completed

** Other SAEs: Haemoptysis due to hospital acquired pneumonia (n=1); Diagnosis of Ca bowel at same admission (n=1)*

*** Other SAE/Re-admissions: Atypical chest pain (n=1); Occlusion of RCA upstream of stent (n=1); Acute stent thrombosis to long stented segment LAD, Orthopnoea, hot/sweaty, chest pain, ST elev, PPCI call (n=1); Failed PCI Urgent CABG organised (n=1); Femoral Artery Aneurysm (n=1); Continued pain right forearm, Episodes of chest pain (n=1); Non cardiac chest pain (n=1)*

**** Other SAE/Re-admissions: Angiography/MRI assessment-elective admission (n=1); AVR and ascending aorta and arch replacement (n=1); Chest pain (n=2); Elective admission-laparoscopic R hemicolectomy (n=1); Angioplasty (staged procedure/day case) (n=1); Staged procedure-angioplasty/vessel PCI (elective) (n=1);*

***** Other SAE/Re-admissions: Admitted with chest pain (n=1); Infective exacerbation of COPD, Chest pain and shortness of breath, Shortness of breath (n=1); Increasing shortness of breath (n=1); Atypical chest pain (n=1); Severe hypertension, Chest pain-stable angina (n=1); Post infarct elective PCI to LAD, Admission with diarrhoea and malaise (n=1); Tired and generally unwell with 2-3d of abdo pain, Abdominal pain-subacute obstruction-Neutropenic, Removal of liver metastasis, Abdominal pain caused by subhepatic obstruction (n=1); Patient fell colar and cuff to right arm, Unwell, confusion, vascular dementia, UTI (n=1); Incidental sigmoid tumour on CT/partial obstruction, Elective admission for sigmoid colectomy (n=1); Excision of basal carcinoma of the left ala (n=1); Right sided facial twitching and blepharospasm, Atrial fibrillation - Elective DC cardioversion (n=1); Off-pump CABG x 1 (LIMA to LAD) - Elective/Waiting (n=1)*

Abbreviations: *DM=Diabetes Mellitus, STEMI=ST-elevation myocardial infarction, NSTEMI=Non-ST-elevation myocardial infarction, SAE=Serious adverse event*

Supplementary Table 12: Details of unexpected serious adverse events

Patient	Event	Description	Reason SAE	Maximum intensity	Relatedness	Group
1	1	Severe hypertension despite multiple drug treatment.	Required hospitalisation	Moderate	Not related	STEMI with DM
	2	Chest pain-Stable angina	Required hospitalisation	Moderate	Not related	STEMI with DM
2	1	Post infarct elective PCI to LAD (Cardiac Angio Coronary Stent x2)	Required hospitalisation	Moderate	Not related	STEMI with DM
	2	Patient admitted with diarrhoea and malaise	Required hospitalisation	Moderate	Not related	STEMI with DM
3	1	Patient was discharged post-MI. Patient was re-admitted with acute stent thrombosis to long stented segment LAD.	Required hospitalisation	Moderate	Not related	STEMI with DM
	2	Admitted to BHI with orthopnoea, hot and sweaty. Chest pain and minor increase in anterior ST elevation.	Required hospitalisation	Moderate	Not related	STEMI with DM
	3	Patient was admitted with chest pain, SOB and an episode of sweating.	Required hospitalisation	Moderate	Not related	STEMI with DM
4	1	Acute Ischaemic Heart Disease - Admission in Royal United Hospital	Is/was life threatening, Required hospitalisation, Prolonged an ongoing hospitalisation	Severe	Not related	STEMI with DM
5	1	Mild atypical chest pain after recent MI and was re-admitted	Required hospitalisation	Moderate	Not related	STEMI, without DM
6	1	Patient had a significant GI ulcer bleed where they dropped their haemoglobin and required 8 units as a transfusion	Prolonged an ongoing hospitalisation	Severe	Not related	STEMI, without DM
7	1	Patient was admitted to Hospital with recurrent left upper chest ache and numbness which radiated to the side of his neck	Required hospitalisation	Moderate	Not related	STEMI, without DM
8	1	Admission to hospital requiring revascularisation of RCA occlusion upstream of stent, implanted.	Required hospitalisation	Moderate	Not related	STEMI, without DM
	2	Planned admission for CABG/aortic valve/root repair/replacement. Procedure performed - AVR and replacement of ascending aorta and arch. Post op VF arrest, cardiac instability requiring inotropic support. Prolonged respiratory wear and tracheostomy. Left innominate vein tied off during surgery.	Required hospitalisation, Prolonged an ongoing hospitalisation	Moderate	Not related	STEMI, without DM
	3	Admission to hospital with shortness of breath.	Required hospitalisation	Moderate	Not related	STEMI, without DM
	4	Admission to hospital - increase SOB and 3/7; presented with asthma attack. Known COPD on home O2.	Required hospitalisation	Moderate	Not related	STEMI, without DM
	5	Admission to hospital with shortness of breath and chest pain.	Required hospitalisation	Moderate	Not related	STEMI, without DM
9	1	Admission with atypical left sided sub mammary pain. Unstable angina.	Required hospitalisation	Moderate	Not related	STEMI, without DM

Patient	Event	Description	Reason SAE	Maximum intensity	Relatedness	Group
10	1	Elective admission for angioplasty to bystander coronary disease	Resulted in persistent or significant disability/incapacity Required hospitalisation	Moderate	Not related	STEMI, without DM
11	1	1cm aneurysm and selling of femoral artery - Right side	Is/was life threatening	Mild	Not related	STEMI, without DM
	2	Admitted with 2 days history of chest pain of very short duration.	Required hospitalisation	Moderate	Not related	STEMI, without DM
12	1	Right sided facial twitching and blepharospasm	Required hospitalisation	Moderate	Not related	STEMI, without DM
13	1	Patient was admitted on with increasing shortness of breath. No chest pain.	Required hospitalisation	Moderate	Not related	NSTEMI, with DM
	2	Failed PCI (patient unable to tolerate procedure). Urgent CABG organised following MRI and Echo assessments. Remained in hospital.	Prolonged an ongoing hospitalisation	Severe	Not related	NSTEMI, with DM
14	1	Angio failed: Need CABG surgery.	Required hospitalisation	Severe	Not related	NSTEMI, with DM
15	1	Admitted with chest pain; acute coronary syndrome without ST elevation.	Required hospitalisation	Moderate	Not related	NSTEMI, without DM
16	1	Elective admission for laparoscopic right hemicolectomy and en-bloc resection of small bowel.	Required hospitalisation	Moderate	Not related	NSTEMI, without DM
	2	Gripey abdominal pain.	Required hospitalisation	Mild	Not related	NSTEMI, without DM
	3	Presenting symptom: abdominal pain. Diagnosis: subacute obstruction and neutropenic fever.	Is/was life threatening, Required hospitalisation	Moderate	Not related	NSTEMI, without DM
	4	Patient developed NSTEMI post-operatively after elective admission for removal of liver metastasis. Chest pain started on 16/06/2012.	Is/was life threatening, Prolonged an ongoing hospitalisation	Severe	Not related	NSTEMI, without DM

Abbreviations: *DM=Diabetes Mellitus, STEMI=ST-elevation myocardial infarction, NSTEMI=Non-ST-elevation myocardial infarction, SAE=Serious adverse events*
